# Nanobodies from camelid mice and llamas neutralize SARS-CoV-2 variants

**DOI:** 10.1038/s41586-021-03676-z

**Published:** 2021-06-07

**Authors:** Jianliang Xu, Kai Xu, Seolkyoung Jung, Andrea Conte, Jenna Lieberman, Frauke Muecksch, Julio Cesar Cetrulo Lorenzi, Solji Park, Fabian Schmidt, Zijun Wang, Yaoxing Huang, Yang Luo, Manoj S. Nair, Pengfei Wang, Jonathan E. Schulz, Lino Tessarollo, Tatsiana Bylund, Gwo-Yu Chuang, Adam S. Olia, Tyler Stephens, I-Ting Teng, Yaroslav Tsybovsky, Tongqing Zhou, Vincent Munster, David D. Ho, Theodora Hatziioannou, Paul D. Bieniasz, Michel C. Nussenzweig, Peter D. Kwong, Rafael Casellas

**Affiliations:** 1 Lymphocyte Nuclear Biology, NIAMS, NIH, Bethesda, MD USA; 2 Vaccine Research Center, NIAID, NIH, Bethesda, MD USA; 3 Laboratory of Retrovirology, The Rockefeller University, New York, NY USA; 4 Laboratory of Molecular Immunology, The Rockefeller University, New York, NY USA; 5 Aaron Diamond AIDS Research Center, Columbia University Vagelos College of Physicians and Surgeons, New York, NY USA; 6 Laboratory of Virology, Division of Intramural Research, NIAID, NIH, Rocky Mountain Laboratories, Hamilton, MT USA; 7 Mouse Cancer Genetics Program, CCR, NCI, NIH, Frederick, MD USA; 8 Electron Microscopy Laboratory, Cancer Research Technology Program, Frederick National Laboratory for Cancer Research sponsored by the National Cancer Institute, Frederick, MD USA; 9 Howard Hughes Medical Institute, The Rockefeller University, New York, NY USA; 10 The NIH Regulome Project, NIH, Bethesda, MD USA; 11 Center for Cancer Research, NCI, NIH, Bethesda, MD USA; 12 Present Address: Department of Veterinary Biosciences, College of Veterinary Medicine, The Ohio State University, Columbus, OH USA

**Keywords:** Antimicrobial responses, Infectious diseases, SARS-CoV-2

## Abstract

Since the start of the COVID-19 pandemic, SARS-CoV-2 has caused millions of deaths worldwide. Although a number of vaccines have been deployed, the continual evolution of the receptor-binding domain (RBD) of the virus has challenged their efficacy. In particular, the emerging variants B.1.1.7, B.1.351 and P.1 (first detected in the UK, South Africa and Brazil, respectively) have compromised the efficacy of sera from patients who have recovered from COVID-19 and immunotherapies that have received emergency use authorization^1–3^. One potential alternative to avert viral escape is the use of camelid VHHs (variable heavy chain domains of heavy chain antibody (also known as nanobodies)), which can recognize epitopes that are often inaccessible to conventional antibodies^4^. Here, we isolate anti-RBD nanobodies from llamas and from mice that we engineered to produce VHHs cloned from alpacas, dromedaries and Bactrian camels. We identified two groups of highly neutralizing nanobodies. Group 1 circumvents antigenic drift by recognizing an RBD region that is highly conserved in coronaviruses but rarely targeted by human antibodies. Group 2 is almost exclusively focused to the RBD–ACE2 interface and does not neutralize SARS-CoV-2 variants that carry E484K or N501Y substitutions. However, nanobodies in group 2 retain full neutralization activity against these variants when expressed as homotrimers, and—to our knowledge—rival the most potent antibodies against SARS-CoV-2 that have been produced to date. These findings suggest that multivalent nanobodies overcome SARS-CoV-2 mutations through two separate mechanisms: enhanced avidity for the ACE2-binding domain and recognition of conserved epitopes that are largely inaccessible to human antibodies. Therefore, although new SARS-CoV-2 mutants will continue to emerge, nanobodies represent promising tools to prevent COVID-19 mortality when vaccines are compromised.

## Abstract (web summary)

Multivalent nanobodies against SARS-CoV-2 from mice engineered to produce camelid nanobodies recognize conserved epitopes that are inaccessible to human antibodies and show promise as a strategy for dealing with viral escape mutations.

## Main

**Fig. 1:**
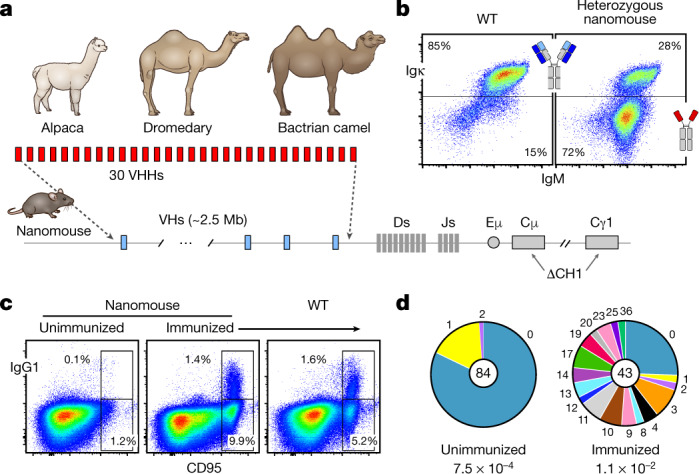
Production of nanomice. **a**, Thirty VHHs selected from alpaca, dromedary and Bactrian camel were inserted via CRISPR–Cas9 in lieu of the 2.5-Mb mouse VH locus. CH1 exons from Cμ and Cγ1 were also deleted to avoid misfolding of the antibody heavy chain. **b**, Flow cytometry analysis of splenic B220^+^ B cells from wild-type (WT) mice or heterozygous nanomice. IgM^+^Igκ^+^ cells express conventional heavy–light chain antibodies, whereas IgM^+^Igκ^−^ cells are mostly Igλ^+^ in wild-type mice (not shown) or single-chain-antibody B cells in nanomice. **c**, Flow cytometry analysis of splenic cells from unimmunized and immunized nanomice and controls stained with CD95 and IgG1. **d**, Pie charts showing VHH somatic hypermutation in unimmunized and immunized nanomice. Pie segments are proportional to the VHH sequences carrying the mutations indicated on the periphery of the chart. The middle circle shows the total number of sequences, and mutation frequency is given below.

**Extended Data Fig. 1:**
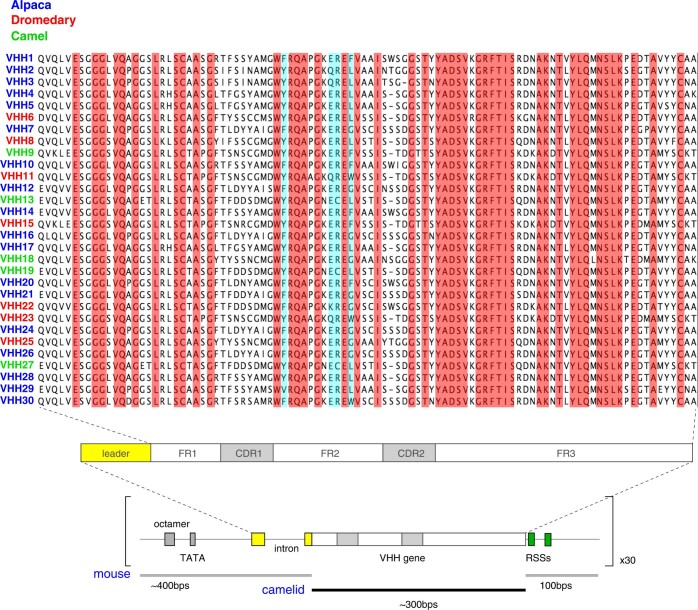
VHH genes used in the array and gene unit assembly. Alignment of the 30 VHH genes, highlighting the 100% amino acid conservation (in red) and the 4 hydrophilic amino acids in framework (FR) 2 (in blue). In VH proteins, these latter four amino acids are hydrophobic and mediate the interaction with light chains. Schematics below show the configuration of VHH gene units, composed of a mouse VH promoter (250 bp, containing the octamer and TATA box); mouse leader exons–intron (about 150 bp) encoding the signal peptide cleaved off during heavy chain processing in the endoplasmic reticulum; the camelid VHH open reading frame (about 300 bp); and mouse downstream sequences (100 bp) containing the recombination signal sequences (RSSs).

In contrast to mouse and human antibody binding domains (which are about 50 kDa in size), camelid VHHs retain full antigen specificity at about 15 kDa. This feature—along with extended complementarity determining regions (CDRs)—enables nanobodies to bind epitopes that are not normally accessible to conventional antibodies^4^, such as conserved viral domains that are often masked by glycan shields. Nanobodies can be readily humanized^5^ and in recent clinical trials they appeared safe and of low immunogenicity^6^. Despite these advantages, nanobodies are not widely used. One reason is that camelids are large animals that are not suitable for academic facilities. There are also few reagents available to isolate antigen-specific memory B cells from immunized camelids^7^. To bypass these hurdles, we sought to produce nanobodies in mice by combining 18 alpaca, 7 dromedary and 5 Bactrian camel VHH genes in a 25-kb insertion cassette (Fig. 1a). Each gene was fused to a VH promoter, leader exons and recombination signal sequences to ensure physiological expression and recombination (Extended Data Fig. 1). Using CRISPR–Cas9, we inserted the VHH cassette in lieu of the VH locus in mouse embryonic stem cells (Fig. 1a).

Camelid nanobodies are expressed only in conjunction with dedicated IgG2 and IgG3, which splice out the CH1 exon during transcription^4^. In conventional antibodies, the hydrophobic surface of CH1 helps to pair heavy and light chain constant domains. To recapitulate this configuration in the mouse genome, we deleted CH1 from both *Ighm* and *Ighg1* in the embryonic stem cells (Fig. 1a). The targeted allele was germline-transmitted from mouse chimeras to F_1_ offspring (hereafter referred to as ‘nanomice’).

**Extended Data Fig. 2:**
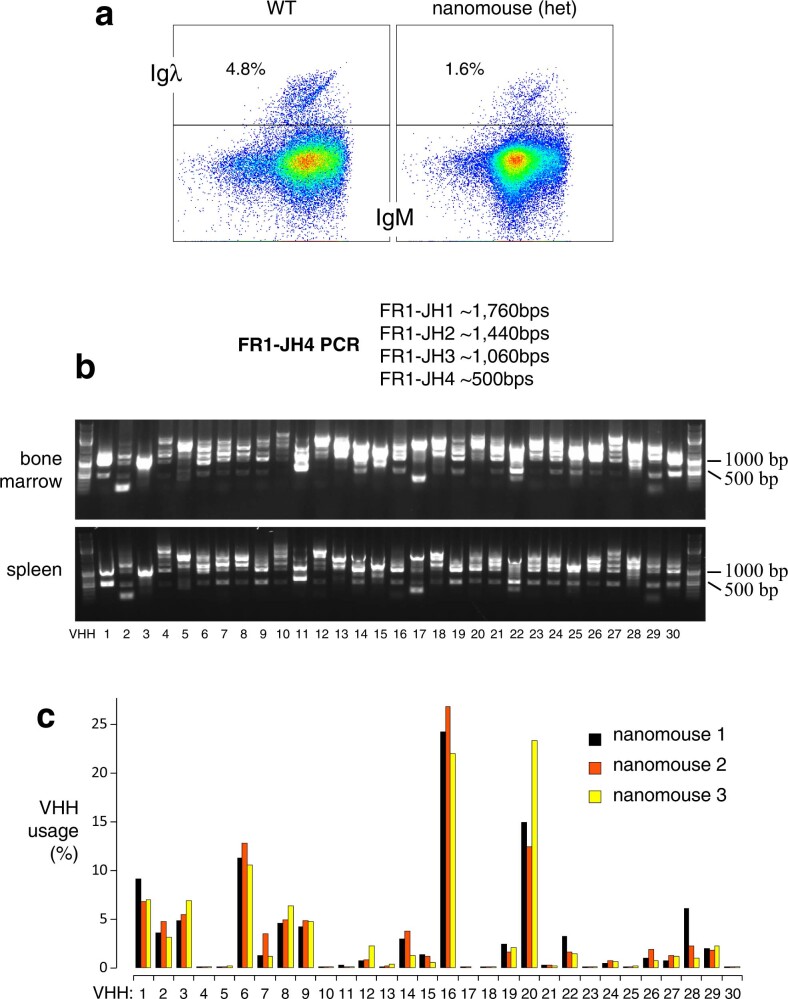
Igλ expression and recombination frequency of VHH genes. **a**, Flow cytometry analysis of Igλ expression in B220^+^IgM^+^ splenic B cells from wild type and heterozygous nanomice. **b**, VHH–DJ recombination was monitored by genomic PCR in bone marrow and spleen samples using an FR1 VHH-specific primer and a second primer downstream of JH4. The expected PCR products for each recombination event between a given VHH and JH1, JH2, JH3 or JH4 are provided. Data are representative of two independent experiments. **c**, Bar graph showing VHH percentage use among splenic B cells in three nanomouse littermates.

As expected, about 85% of splenic B220^+^ B cells in wild-type mice were IgM^+^Igκ^+^ (Fig. 1b, left). By contrast, 72% of splenic B220^+^ B cells in heterozygous nanomice displayed an IgM^+^Igκ^−^ phenotype (Fig. 1b, right) and of these less than 2% were IgM^+^Igλ^+^ (Extended Data Fig. 2a), which implies that a large fraction of nanomouse B cells develop expressing single-chain antibodies. We confirmed this observation by amplifying VHH–DJ joining events using gene-specific primers. We found that all 30 VHHs were recombined to downstream JHs in bone marrow and spleen samples (Extended Data Fig. 2b). We performed a deep-sequencing analysis, which confirmed that all VHH genes undergo V(D)J recombination and are thus potentially available for expansion during the immune response (Extended Data Fig. 2c).

**Extended Data Fig. 3:**
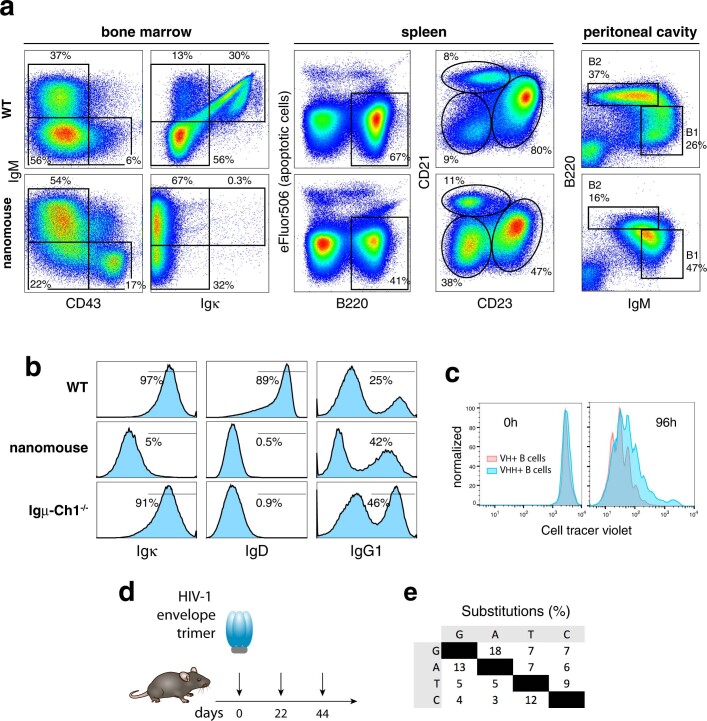
B cell development in nanomice. **a**, Flow cytometry analysis of bone marrow (left two columns), spleen (third and fourth columns) and peritoneal cavity B cells (last column) in wild-type controls and nanomice. First column shows the percentage of B220-gated CD43^+^IgM^−^ proB and CD43^-^IgM^+^ immature B cells. Second column shows percentage of IgM and Igκ within the B220-gated population. Third column denotes the total number of B220^+^ B cells in the spleen. The *y* axis shows viability staining with eFluor506 (eBiosciences). The fourth column shows the percentage of B220-gated CD23^low^CD21^low^ immature, CD23^high^CD21^low^ follicular, and CD23^low^CD21^high^ marginal-zone splenic, cells in the two strains. The last column shows the percentage of B1 (IgM^high^B220^low^) and B2 (IgM^low^B220^high^) cells in the peritoneal cavity. Examples of gating for bone marrow and splenic B cells is provided in Extended Data Fig. 10d. **b**, Histograms depicting the percentage of Igκ (left), IgD (middle) and IgG1 (right row) in wild-type, nanomice, and *Ighm*-CH1^−/−^ mice. The latter measured in ex vivo cultures treated with LPS, IL-4 and anti-CD180. Population gates are represented with a line and the percentage of total cells is provided. **c**, Proliferation assay of nanomouse and control B cells cultured for 96 h with LPS, IL-4 and anti-CD180. **d**, Immunization regimen. Nanomice were immunized with 50 μg HIV-1 envelope trimer at the indicated dates. **e**, Per cent nucleotide substitutions (adjusted for base composition) observed in nanobodies isolated from immunized nanomice. Phage library was selected for binding to HIV-1 envelope trimer.

In VHH-homozygous nanomice, the B cell compartment was largely normal and displayed all developmental stages, including B1, B2 and marginal-zone B cells (Extended Data Fig. 3a). One difference was an increased number of IgM^+^ transitional and immature B cells in the bone marrow and spleen, respectively, indicative of enhanced selection as cells transition from the short- to the long-lived CD23^high^CD21^low^ compartment, which in nanomice was reduced 1.7-fold relative to wild-type mice (Extended Data Fig. 3a). Another distinct feature was the absence of IgD (Extended Data Fig. 3b). This phenotype probably results from differential mRNA splicing owing to CH1 deletion at *Ighm*, because IgD was also absent in mice that are homozygous for deletion of the *Ighm* CH1 only (in which VHs and *Ighg1* CH1 are intact) (Extended Data Fig. 3b). Taken together, these data show that mouse B cells can mature expressing single-chain antibodies.

## Activation and hypermutation in nanomice

To probe activation, splenic B cells were isolated and cultured in the presence of lipopolysaccharide and interleukin-4. Under these conditions, VHH-expressing cells underwent proliferation and switch recombination to IgG1 (Extended Data Fig. 3b, c). To examine activation in vivo, we performed intraperitoneal immunizations with keyhole limpet haemocyanin. Twelve days after immunization, nanomice showed numbers of B220^+^CD95^high^IgG1^+^ germinal-centre B cells equivalent to those of controls (Fig. 1c).

To study affinity maturation against a specific antigen, we immunized nanomice with human immunodeficiency virus-1 (HIV-1) envelope trimer (BG505 DS-SOSIP)^8^ (Extended Data Fig. 3d). Hypermutation of VHH genes was increased relative to unimmunized controls (1.1 × 10^−2^ versus 7.5 × 10^−4^) (Fig. 1d). The mutation spectra revealed an enrichment in G-to-A and C-to-T transitions (Extended Data Fig. 3e), consistent with activation-induced cytidine deaminase catalysis^9^.

**Extended Data Fig. 4:**
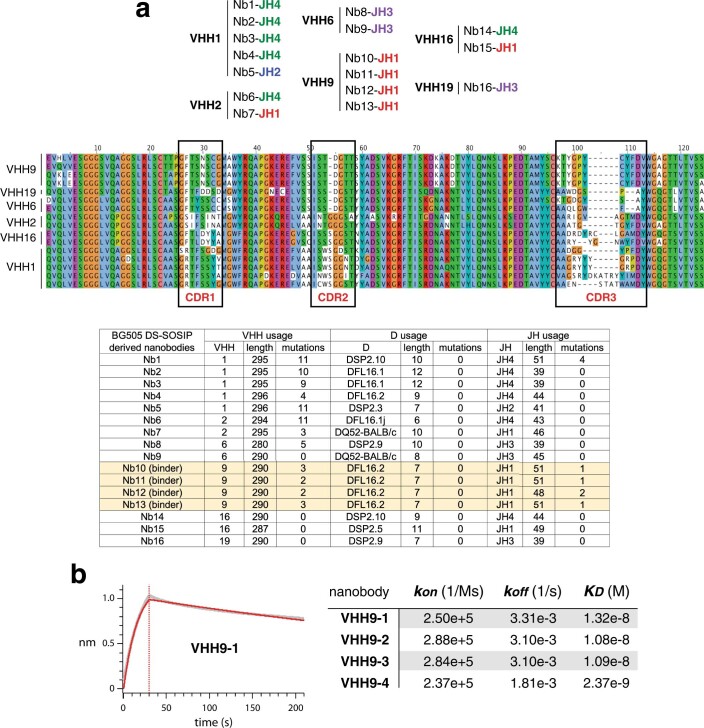
Nanomouse immune response to HIV-1 envelope trimer. **a**, Top table shows nanobody and JH use for the 16 VHH clones isolated from immunized nanomice. Middle graph shows protein alignment for VHHs isolated from HIV-1 trimer immunized nanomice. CDRs are boxed. Bottom table shows hypermutation profiles for VHH, D and J domains of selected nanobodies. **b**, Left, BLI analysis of BG505 DS-SOSIP binding to immobilized VHH9-1. Red trace represents the raw data, and the kinetic fit is shown in grey underneath. Right, table showing the kinetic constants for association (*k*_on_), dissociation (*k*_off_) and equilibrium (*K*_D_) for all four VHH9 nanobody variants.

To measure the antibody response against BG505 DS-SOSIP, we characterized 16 nanobodies that were enriched for HIV-1 trimer recognition. Sequence analysis showed CDR3s to be highly diverse in this group in terms of JH use, mutations and size (9–16 amino acids) (Extended Data Fig. 4a). To measure binding kinetics, we applied biolayer interferometry. The analysis identified four VHH9 variants, which displayed dissociation constants (*K*_D_s) that ranged from 2 to 13 nM—demonstrating that they represent high-affinity binders (Extended Data Fig. 4b, Supplementary Table 1). We conclude that mouse B cells that express single-chain antibodies can undergo affinity maturation and produce highly specific nanobodies upon immunization.

## SARS-CoV-2 neutralizing nanobodies

**Fig. 2:**
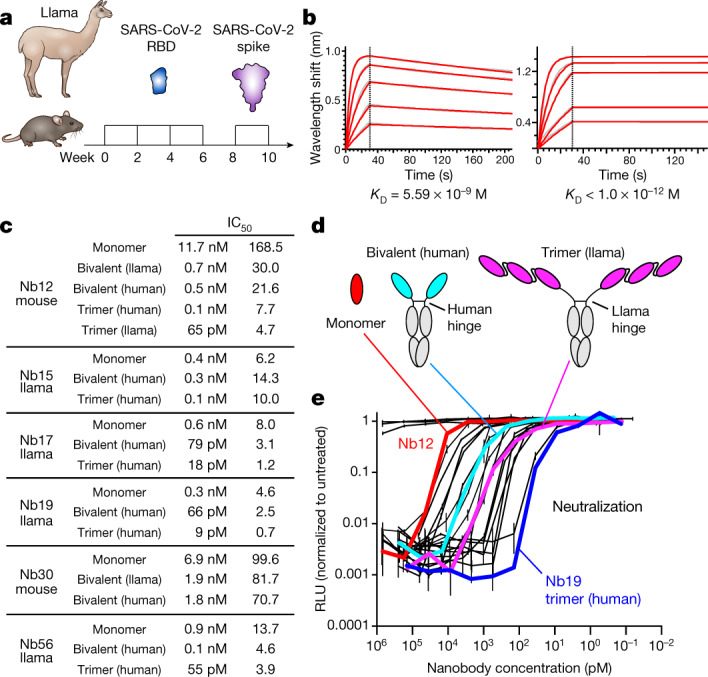
Isolation of nanobodies against SARS-CoV-2. **a**, Immunization of llama and nanomice to obtain high-affinity nanobodies against SARS-CoV-2 RBD. **b**, Biolayer interferometry (BLI) analysis of difference concentrations of Nb17 monomer (left) and trimer (right) binding to immobilized RBD. Red trace represents the raw data; the kinetic fit is shown in grey underneath. Equilibrium (*K*_D_) constants are provided. **c**, Table summarizing pseudovirus neutralization potency (IC_50_) of selected nanobodies. Values are provided in molarity (left) or as ng ml^−1^ (right). **d**, Diagrams showing nanobodies used in neutralization assays as monomers, bivalent or trimers (the last two fused to human IgG1 Fc via the human or llama IgG2a hinge domain). **e**, Neutralization of SARS-CoV-2 pseudovirus by the 20 nanobodies shown in **c**. Nb12 monomer (red), bivalent (cyan) and trimer (magenta), as well as Nb19 trimer (blue), are highlighted. Data are representative of two independent experiments and the error bars are mean ± s.d. of triplicates.

**Extended Data Fig. 5:**
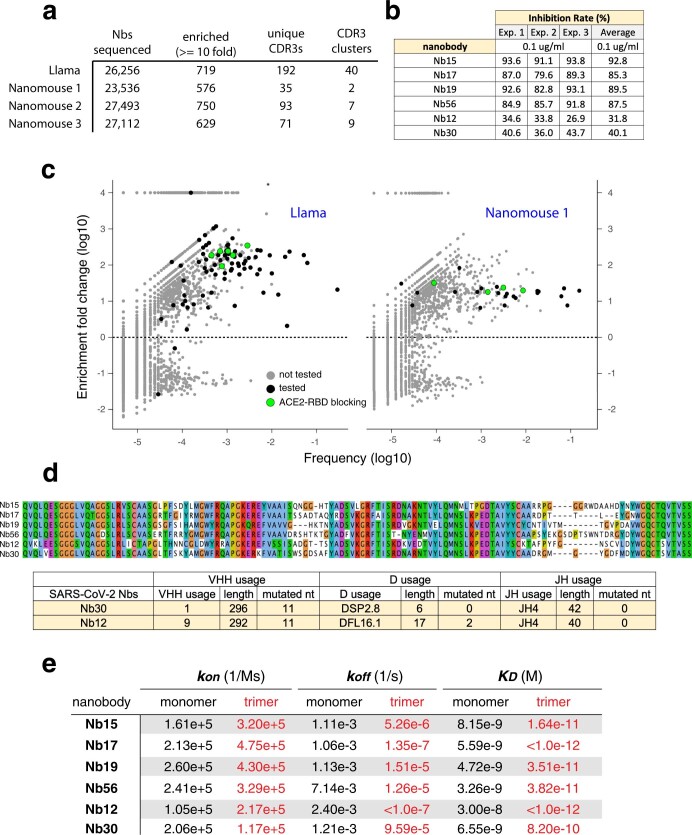
Isolation of anti-SARS-CoV-2 RBD nanobodies. **a**, Table indicating (1) the total number of unique nanobody genes identified from llama and three nanomice phage display libraries following selection for RBD binding; (2) the number of nanobodies enriched at least tenfold after selection; (3) the number of nanobodies with a unique CRD3; and (4) the different clusters of nanobodies that share similar CDR3 s (with no more than 2 amino acid differences). **b**, Table showing in vitro neutralization results for the six leading nanobodies using the sVNT kit of GenScript. **c**, Dot plot depicting the extent of enrichment (*y* axis) and frequency (*x* axis) of unique nanobodies after RBD selection of llama (left) or nanomouse 1 (right) libraries. Green circles represent nanobodies that block ACE2–RBD interactions in vitro, black circles are nanobodies that do not efficiently block ACE2–RBD interactions, and grey dots represent untested nanobodies. **d**, Top graph shows protein alignment of the six nanobodies isolated from llama and nanomice immunized with SARS-CoV-2 spike and RBD. Bottom table shows detailed information of the VHH, D and J domains of nanomouse nanobodies. **e**, Table depicting equilibrium (*K*_D_), association (*K*_on_) and dissociation (*K*_off_) constants obtained for each nanobody as a monomer (black) or trimer (red) form.

We next sought to produce neutralizing nanobodies against SARS-CoV-2. To this end, we immunized three nanomice and one llama with RBD and the stabilized prefusion spike of SARS-CoV-2 (Fig. 2a). We isolated peripheral blood mononuclear cells after immunization, and amplified and cloned VHHs into a phagemid vector. Following phage display, we enriched RBD-specific nanobodies using an enzyme-linked immunosorbent assay-based binding screen. Our deep-sequencing analysis identified, on average, 26,000 nanobody variants per library, which represents a total of 192 and 199 unique CDR3s for llama and nanomice, respectively (Extended Data Fig. 5a). We then clustered the nanobodies by CDR3s (Methods), isolated representative clones from each subgroup and tested them for blocking RBD binding to the ACE2 receptor in vitro^10^. We selected six llama and six nanomouse nanobodies using this method.

To refine the list of candidates, we measured RBD-binding affinity by biolayer interferometry. This analysis identified 4 nanobodies from llama (designed nanobody (Nb) 15, Nb17, Nb19 and Nb56) and 2 nanobodies from nanomouse (Nb12 and Nb30) with dissociation constants below 30 nM (Fig. 2b, Extended Data Fig. 5b–d, Supplementary Table 1). The off-rate varied from 7.1 × 10^−3^ s^−1^ to 1.1 × 10^−3^ s^−1^, demonstrating slow dissociation for all nanobodies (Extended Data Fig. 5e). We next explored neutralization in vitro using lentiviral particles pseudotyped with the SARS-CoV-2 spike^11^. The nanobody monomers displayed nanomolar and sub-nanomolar half-maximal inhibitory concentration (IC_50_) values that ranged from 11.7 nM (168.5 ng ml^−1^) for Nb12 to 0.335 nM (4.6 ng ml^−1^) for Nb19 (Fig. 2c).

A crucial advantage of nanobodies over conventional antibodies is that they can be easily assembled into multimers, which often results in marked avidity^12,13^. To explore this property, we fused nanobodies as trimers using flexible GGGGS(×3) linkers and connected them to human IgG1 Fc via the human hinge domain or its much longer, flexible llama counterpart (Fig. 2d). We also created bivalent antibodies by fusing two VHHs to IgG1 Fc (Fig. 2d). We found that neutralization increased with the number of linked monomers, from 3-fold for Nb15 to 180-fold for Nb12 (Fig. 2c, e). Notably, the four most potent multimeric nanobodies (Nb12, Nb17, Nb19 and Nb56) reached IC_50_ values in the picomolar range (from 65 to 9 pM) (Fig. 2c, e). To our knowledge, these values rank among the best reported to date for anti-SARS-CoV-2 nanobodies^14^.

## Nanobodies overcome SARS-CoV-2 mutants

With the worldwide spread of SARS-CoV-2, several variants that carry RBD mutations have emerged that increase transmissibility or allow escape from antibody neutralization. Of particular interest is the B.1.1.7 variant (which contains an N501Y substitution) that caused an upsurge in COVID-19 cases in the UK^15^. A second variant of concern is B.1.351, which combines N501Y with two additional RBD substitutions (K417N and E484K). P.1, a third variant that spread rapidly in Brazil, shows changes similar to those of B.1.1.7 and B.1.351: N501Y, K417T and E484K^16^. All of these mutations have been shown to reduce the efficacy of serum antibodies elicited by the Moderna and Pfizer–BioNTech vaccines^1,2^.

**Fig. 3:**
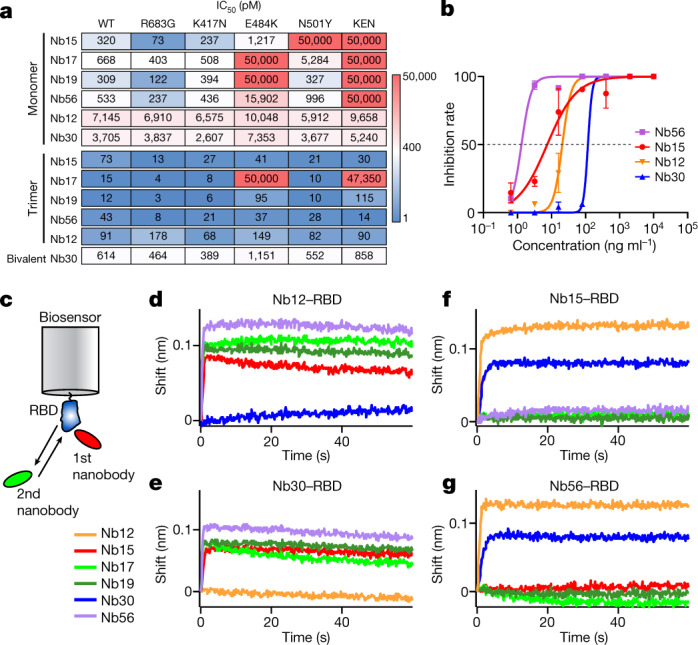
Neutralization of wild-type and mutant SARS-CoV-2. **a**, Neutralization assays (IC_50_ values) of pseudoviruses carrying wild-type or mutant SARS-CoV-2 spike. Colour gradient indicates values ranging from 0 (blue) to 50,000 pM (red). Pseudotyped viruses containing E484K or K417N, E484K and N501Y (KEN) also contain the R683G substitution. **b**, Neutralization assay showing the sensitivity of SARS-CoV-2 B.1.351 to different concentrations of trivalent Nb15, Nb56 and Nb12, and bivalent Nb30. Data are representative of two independent experiments and the error bars are mean ± s.d. of triplicates. **c**, Schematics summarizing the BLI competition assay, in which nanobody–RBD immunocomplexes attached to a biosensor are incubated with different nanobodies to measure binding. **d**–**g**, Binding of nanobodies to Nb12–RBD (**d**), Nb30–RBD (**e**), Nb15–RBD (**f**) and Nb56–RBD (**g**) immunocomplexes.

**Extended Data Fig. 6:**
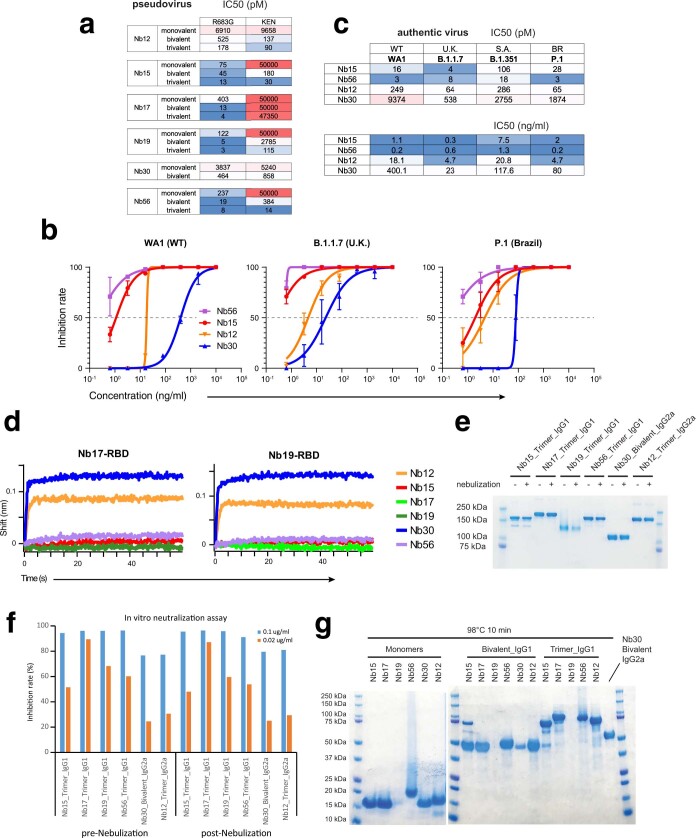
Neutralization of pseudo and authentic SARS-CoV-2 viruses. **a**, Comparison of neutralization activities of leading nanobodies in monovalent, bivalent or trivalent form (results for monovalent and trivalent reproduced from Fig. 3a). **b**, Neutralization assays for wild-type (WA1) and SARS-CoV-2 variants B.1.1.7 and P.1 for trivalent Nb56, Nb15, Nb12 and bivalent Nb30. Data are representative of two independent experiments. Data are mean ± s.d. of triplicates. **c**, IC_50_ neutralization values for experiments shown in Fig. 3b and **b**. Top table values are in pM, lower table values are in ng ml^−1^. **d**, Related to Fig. 3c–f. Immunocomplexes used were Nb17–RBD (left) and Nb19–RBD (right). **e**, Coomassie staining showing nanobody integrity following nebulization. With the exception of Nb30 (bivalent), all nanobodies were fused to Fcs as trimers. Data are representative of two independent experiments. **f**, Bar graph showing in vitro neutralization (percentage) of RBD–ACE2 interactions by the different trivalent nanobody (bivalent for Nb30) before and after nebulization at two different concentrations (0.1 μg ml^−1^ (blue) and 0.02 μg ml^−1^ (orange)). **g**, Coomassie staining showing integrity of nanobody monomers (left) or multimers (right) following heat treatment (98 °C for 10 min). Data are representative of two independent experiments.

We first explored whether our leading nanobodies could neutralize virus pseudotyped with SARS-CoV-2 spike carrying the RBD mutations. The R683G substitution, which increases infectivity in vitro^17^ was included as a control. In contrast to their efficacy against the wild-type virus, Nb17, Nb19 and Nb56 were unable to neutralize viruses carrying the E484K substitution alone or in combination with K417N and N501Y (Fig. 3a). Similarly, Nb15 was ineffective against N501Y. However, with the exception of Nb17, the nanobodies all remained highly potent binders and neutralizers in bivalent or trivalent forms (Fig. 3a, Extended Data Figs. 5e, 6a). In the case of Nb15 and Nb56 trimers, IC_50_ values reached 30 pM and 14 pM, respectively. Thus, the E484K and N501Y substitutions enable viral escape from monomeric, but not multimeric, nanobodies.

In contrast to llama nanobodies, the neutralization potencies of nanomouse Nb12 and Nb30 were largely unaltered by RBD mutations (Fig. 3a), which suggests that they recognize a region that is different from the receptor-binding motif. To explore whether multimeric nanobodies function against authentic virus, we repeated the neutralization assay with trivalent Nb15, Nb56 and Nb12, and bivalent Nb30, using SARS-CoV-2 WA1 and the B1.1.7, B1.351 and P.1 variants. The results closely recapitulated the pseudovirus findings, showing neutralization of wild type and the three variants by all four nanobodies (Fig. 3b, Extended Data Fig. 6b). Of note, these nanobodies were most effective against the B1.1.7 variant, with IC_50_ values that ranged between 4 pM (for Nb15) and 538 pM (for Nb30), and were relatively less effective against the B.1.351 variant, showing a range of 18 pM (for Nb56) to 2,755 pM (for Nb30) (Extended Data Fig. 6c). Neutralization of the P.1 variant was intermediate (Extended Data Fig. 6b, c).

The fact that llama and nanomouse nanobodies are differentially affected by the variants suggests that they recognize different RBD epitopes. To test this idea, we applied biolayer interferometry using a preformed nanobody–RBD immunocomplex that was incubated with a second nanobody (Fig. 3c). We found that all four llama nanobodies, but not Nb30, could bind the Nb12–RBD immunocomplex (Fig. 3d). Similarly, Nb30–RBD interfered with Nb12 binding, whereas llama nanobodies bound freely to it (Fig. 3e). At the same time, Nb12 and Nb30 recognized all combinations of llama nanobody–RBD complexes, whereas llama nanobodies could not (Fig. 3f, g, Extended Data Fig. 6d). Thus, nanomouse and llama nanobodies recognize two distinct neutralizing RBD regions.

As is often the case with single-chain antibodies, both llama and nanomouse nanobodies were largely thermostable and could be aerosolized with commercially available mesh nebulizers without losing neutralization activity (Extended Data Fig. 6e–g).

## Nanobody structures

**Extended Data Fig. 7:**
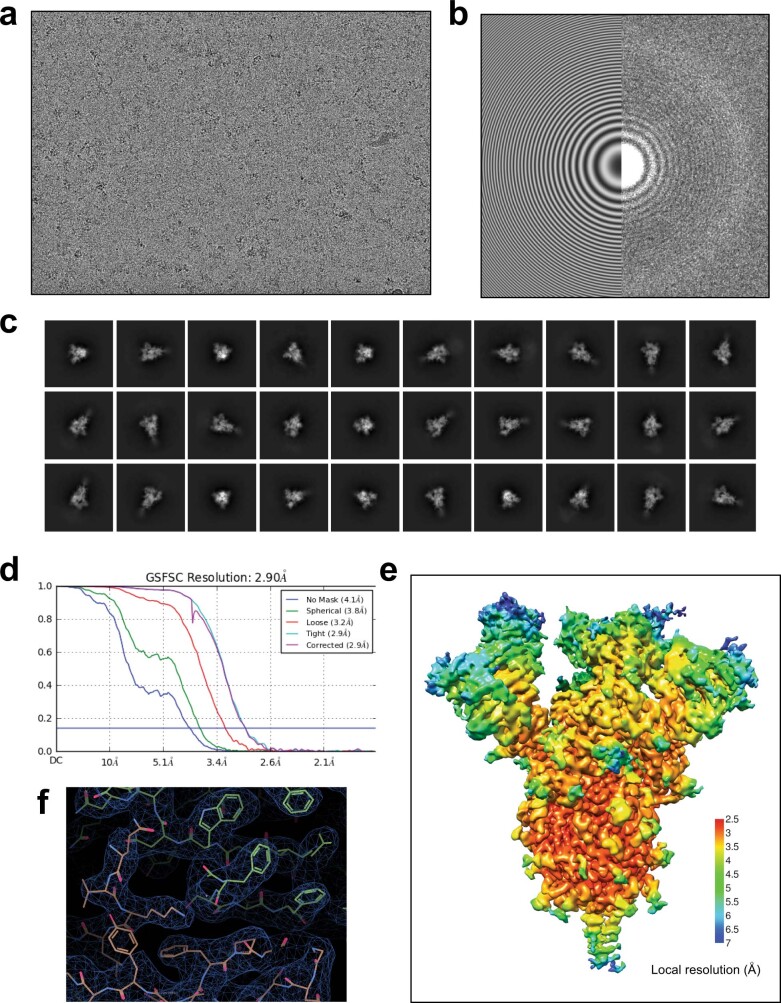
Cryo-EM data processing and validation for Nb12–spike complex. **a**, A representative cryo-EM micrograph showing Nb12–spike complex embedded in vitreous ice. **b**, A contrast-transfer function (CTF) fit of the micrograph. **c**, Representative 2D average classes. **d**, Overall resolution estimation (Fourier shell correlation (FSC) of 0.143). **e**, Local resolution estimation of the cryo-EM map. **f**, Cryo-EM density and models for an interface region between RBD and Nb12 after local refinement.

**Extended Data Fig. 8:**
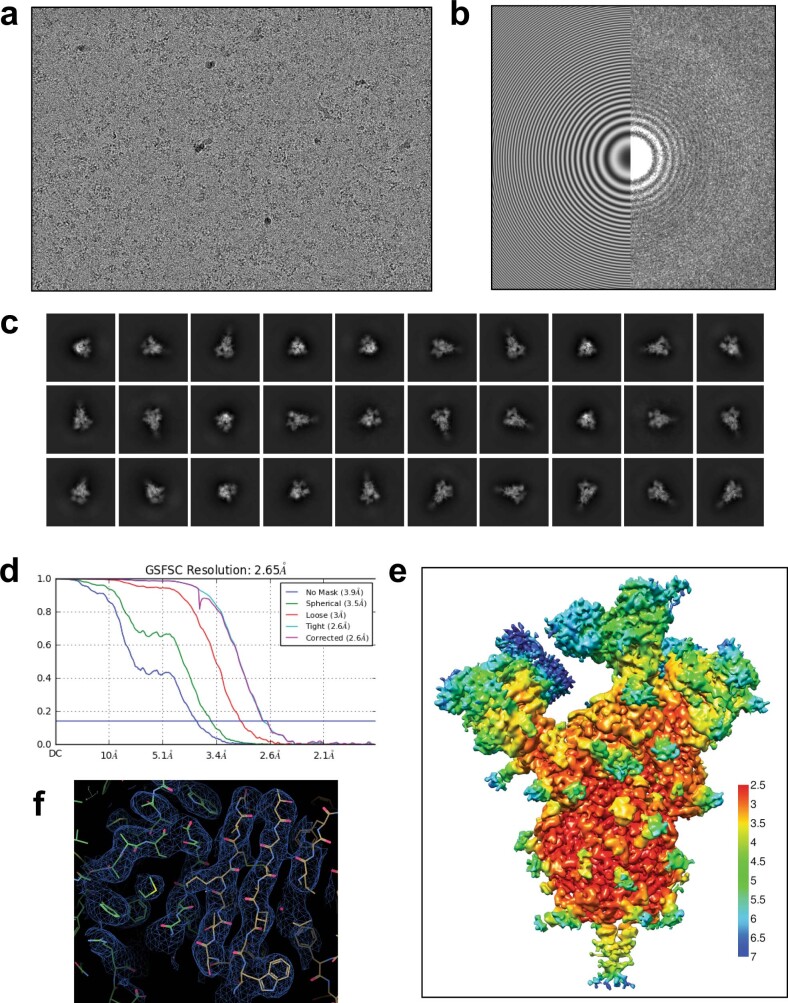
Cryo-EM data processing and validation for Nb30–spike complex. **a**, A representative cryo-EM micrograph showing Nb30–spike complex embedded in vitreous ice. **b**, A CTF fit of the micrograph. **c**, Representative 2D average classes. **d**, Overall resolution estimation (FSC of 0.143). **e**, Local resolution estimation of the cryo-EM map. **f**, Cryo-EM density and models for an interface region between RBD and Nb30 after local refinement.

To define the region bound by nanomouse nanobodies, we collected single-particle cryo-electron microscopy data on a Titian Krios for Nb12 and Nb30 in complex with HexaPro^10^, a prefusion construct of the SARS-CoV-2 spike (Extended Data Figs. 7, 8, Supplementary Table 2). In both cases, we used particle subtraction, classification and local refinement to enhance the resolution of the nanobody–spike interface.

**Fig. 4:**
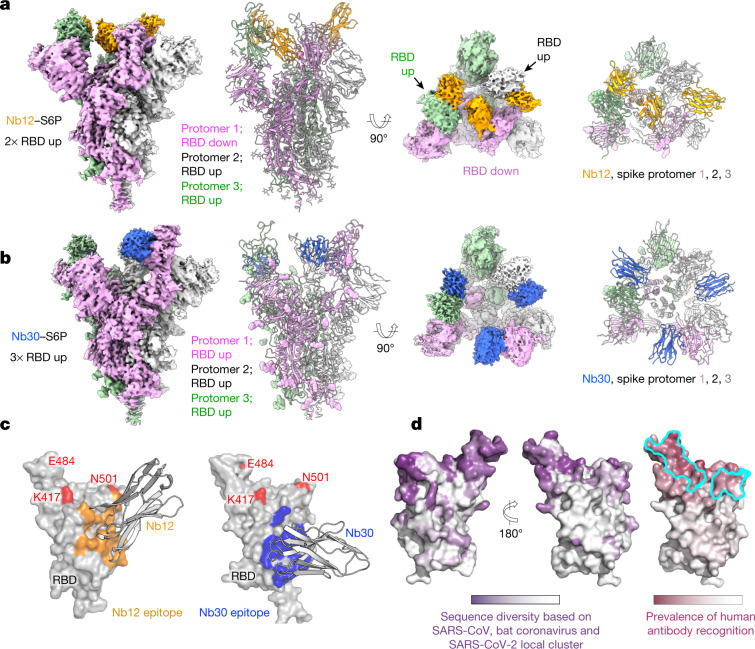
Structures of leading nanobodies in complex with SARS-CoV-2 spike. **a**, Cryo-electron microscopy structure of Nb12 in complex with SARS-CoV-2 spike. **b**, As in **a**, for Nb30. **c**, Interface between nanobodies and spike. **d**, Surface properties of RBD, including sequence diversity (dark purple indicates diversity among sarbecoviruses), and prevalence of RBD-recognized regions by human antibodies (dark raspberry indicates high prevalence) and binding site for ACE2 (cyan).

**Extended Data Fig. 9:**
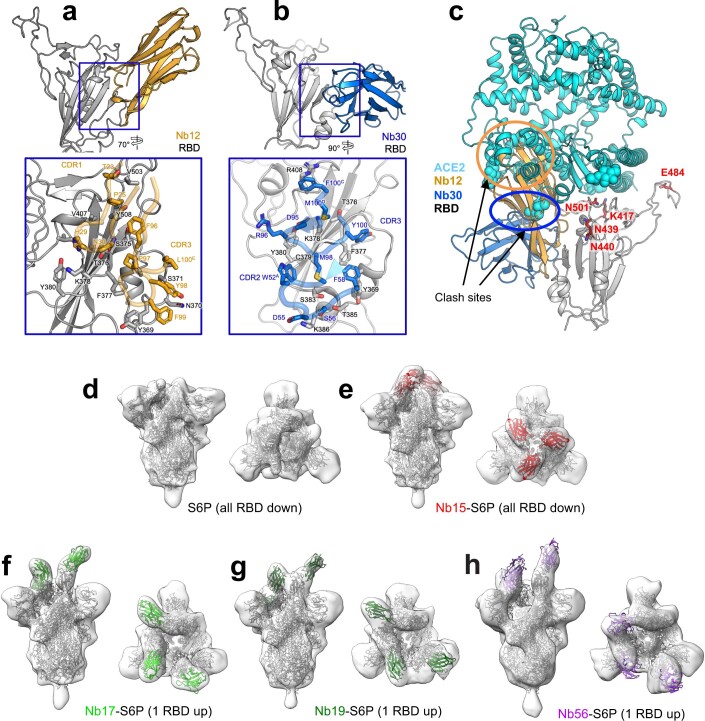
Structural analysis of nanomice and llama nanobody interface with the SARS-CoV-2 spike. **a**, Structure of Nb12 and RBD region (inset, interface between Nb12 and RBD with contact resides shown in stick representation). **b**, Structure of Nb30 and RBD region (inset, interface between Nb30 and RBD with contact resides shown as stick representation). **c**, Cryo-EM defined structures of nanomouse nanobodies recognize regions on RBD distal from residues 417, 484 and 501 (affected by mutations in emerging variants). **d**, SARS-CoV-2 spike (HexaPro) structure in two perpendicular views. **e**, Spike–Nb15 (red) complex structure in two perpendicular views. **f**, Spike–Nb17 (light green) complex structure in two perpendicular views. **g**, Spike–Nb19 (dark green) complex structure in two perpendicular views. **h**, Spike–Nb56 (purple) complex structure in two perpendicular views.

The structure of the Nb12–spike complex revealed Nb12 to induce a two-RBD-up, one-RBD-down spike conformation, with Nb12 recognizing a region towards the middle of the RBD, outside of the ACE2-binding region and distal from the residues (417, 484 and 501) affected in emerging variants of concern (Fig. 4a, Extended Data Fig. 9a). The structure of the Nb30–spike complex revealed Nb30 to induce a three-RBD-up conformation, with Nb30 recognizing a region at the opposite end of RBD from the ACE2-binding motif and residues affected by escape mutations (Fig. 4b, Extended Data Fig. 9b).

To understand how the two nanomouse nanobodies neutralized despite recognizing surfaces outside the ACE2-binding domain, we superimposed the structure of the ACE2–RBD complex^18–20^ with those of Nb12 and Nb30 (Extended Data Fig. 9c). We observed a substantial portion of Nb12 domain clashing with ACE2, which indicates that Nb12 and ACE2 binding are sterically incompatible. With Nb30, we observed a subtler clash with glycan N322 on ACE2, which nonetheless also indicated that Nb30 and ACE2 binding are sterically incompatible.

To obtain a structural understanding of the neutralizing regions recognized by nanomouse and llama nanobodies, we also determined 3D negative-stain electron microscopy reconstructions of each of the nanobodies in complex with HexaPro. These reconstructions revealed that llama nanobodies uniformly target the ACE2-binding interface, with Nb17, Nb19 and Nb56 inducing a one-RBD-up conformation, whereas Nb15 associates with all-RBD-down spikes (Extended Data Fig. 9d–h). By contrast, both nanomouse Nb12 and Nb30 recognize RBD at a surface outside the ACE2-binding site (Fig. 4c).

## RBD regions recognized by mouse nanobodies

**Extended Data Fig. 10:**
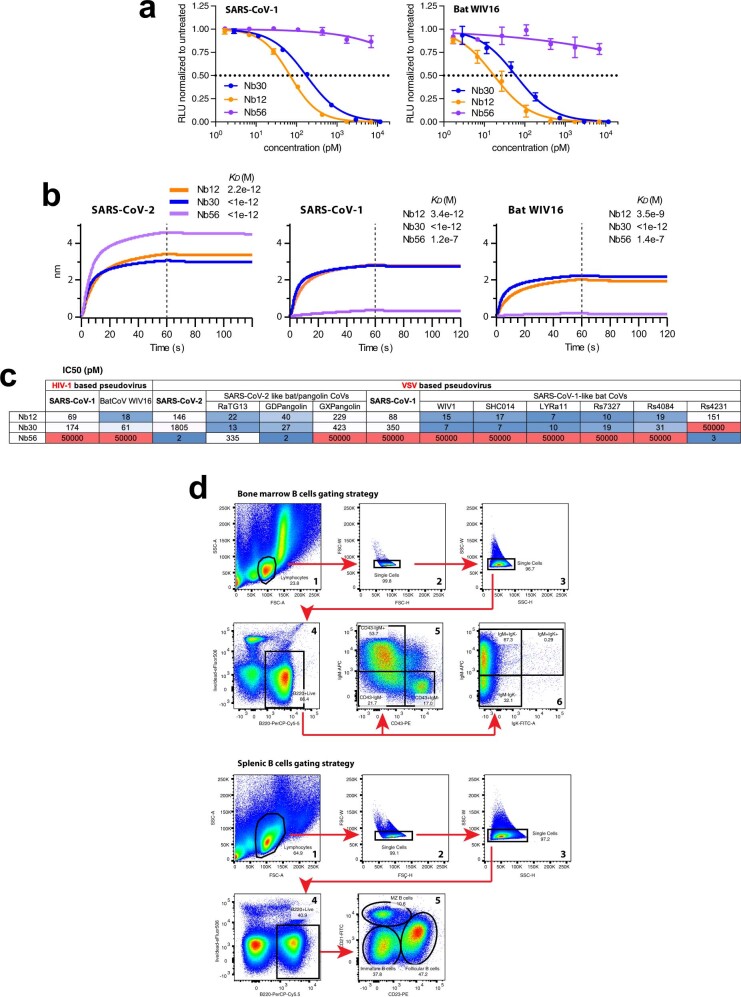
Binding and neutralization of sarbecoviruses by nanomouse nanobodies and gating strategy for nanomouse and wild-type B cells. **a**, Neutralization using trivalent Nb56 and Nb12, and bivalent Nb30, against pseudoviruses carrying SARS-CoV (left) or bat coronavirus WIV16 (right) spikes. Data are representative of two independent experiments and the error bars are mean ± s.d. of triplicates. **b**, BLI analysis of trivalent Nb56 and Nb12, and bivalent Nb30, binding to immobilized RBD from SARS-CoV-2 (left), SARS-CoV (middle) and bat coronavirus WIV16 (right). Equilibrium (*K*_D_) constants are provided. **c**, IC_50_ (pM) values for neutralization in culture assays, showing the sensitivity of HIV-1 and VSV pseudotyped viruses containing 13 sarbecoviral spike proteins. **d**, Analysis of bone marrow (top) or splenic (bottom) B cells was done by gating lymphocytes (first plot), avoiding aggregates (plots 2 and 3), B220^+^apoptotic^−^ gaiting (plot 4), and visualization with cell-surface makers as indicated (plots 5 and 6).

To provide insight into the prevalence of regions on RBD recognized by nanomouse versus human antibodies, we superimposed 51 RBD-directed human neutralizing antibodies in the Protein Data Bank and quantified the recognition prevalence at the residue level (Fig. 4d, Supplementary Table 3). Although recognition extended over much of the RBD, the prevalence of human antibody recognition was much higher in the ACE2-binding region in which the residues affected by emerging mutations reside. By contrast, the regions recognized by Nb12 and Nb30 were conserved in sarbecoviruses and displayed a substantially lower prevalence of human antibody recognition. The epitope of Nb12 overlaps considerably with those recognized by previous nanobodies specific for SARS-CoV^21^ and SARS-CoV-2^13,22^, which raises the possibility that these nanobodies might also block the new SARS-CoV-2 variants. However, the Nb30 binding footprint is farther away from the ACE2 RBD motif and covers a surface area that is 79% conserved among sarbecoviruses, including SARS-CoV, SARS-CoV-2 and bat coronaviruses (compared to 54% for Nb12 and, on average, 23% for human antibodies) (Fig. 4c, d, Supplementary Table 3). Consistent with these findings, we found that Nb12 and Nb30 bind to the RBD of SARS-CoV and of the bat coronavirus WIV16 and neutralize HIV-1-based pseudoviruses that carry their spike, whereas Nb56 does not (Extended Data Fig. 10a, b). We observed similar neutralization patterns with vesicular stomatitis virus-based pseudoviruses carrying spike proteins from pangolin and six additional bat coronaviruses (Extended Data Fig. 10c). We conclude that nanomouse VHHs circumvent RBD antigenic drift by recognizing a sarbecoviral conserved region outside the ACE2-binding motif.

## Discussion

A key contribution of our study is the creation of nanobody-producing mice. Previous work has explored the transgenic expression of a limited number of llama VHHs^23^. In our model, the 30 VHHs replace the entire VH domain, which leads to physiological recombination and selection during ontogeny. Although our nanomice are capable of producing high-affinity nanobodies, they can be improved further by increasing the number of available VHHs. This could be done by engineering a second allele carrying VHHs from llamas, vicuñas and guanacos (the three camelids that are not represented in our insertion cassette). We anticipate that this and similar improvements in animal models will help to popularize the development of nanobodies against infectious diseases or for basic applications.

As a proof of principle, we used nanomice to produce highly specific nanobodies against SARS-CoV-2 RBD. To date, numerous monoclonal antibodies isolated from patients with COVID-19 or from humanized mice have been shown to block the RBD–ACE2 interface. Unsurprisingly, immunotherapies that involve such antibodies are vulnerable to escape variants that carry mutations at or around the ACE2-binding motif^1–3^. The anti-RBD nanobodies we isolated overcome this limitation in two important ways. First, similar to human antibodies, llama nanobodies (Nb15 and Nb56) hinder ACE2 binding to the spike of the original virus, but they are ineffective against viruses that carry E484K or N501Y substitutions. However, in multimeric form, these nanobodies overcome the block and display marked neutralization potency. This reversal is probably the result of increased avidity for the trimeric spike, or possibly the simultaneous cross-linking of multiple spikes on the viral membrane. Another possibility is that trimers occlude ACE2 access to the RBD. Second, nanobodies isolated from nanomice (Nb12 and Nb30) associate with an RBD region that is highly conserved among sarbecoviruses^21^, but remains inaccessible to most human antibodies. As this region lies outside the ACE2-binding motif, nanobody–RBD contacts are unaffected by the E484K or N501Y substitutions. Importantly, even though the conserved domain does not overlap with the ACE2-binding motif, our structural studies suggest that nanobodies of this class sterically interfere with ACE2–RBD associations. On the basis of these features, we propose that our leading nanobodies may provide valuable tools for passive immunotherapy or pulmonary delivery against current or future SARS-CoV-2 variants of concern.

## Methods

### Data reporting

No statistical methods were used to predetermine sample size. The experiments were not randomized and the investigators were not blinded to allocation during experiments and outcome assessment.

### Construction of exchange-cassette and VHH minigene

To replace the entire mouse VH locus (mm10, chromosome 12: 113,567,224–116,010,427) we assembled a targeting vector (pLH28-exchange-cassette) and a VHH minigene. The targeting vector was built by inserting a selection cassette composed of pEF1a-Puro-TK-2A-EGFP in between wtLoxP and LoxP257 sites within the pLH28 exchange vector^24^. As homology arms, 1-kb and 0.8-kb fragments flanking the VH deletion domain were cloned 5′ and 3′ of the LoxP sites. To build the VHH minigene, VHH genes (18 from alpaca, 7 from dromedary and 5 from Bactrian camel) were selected on the basis of published sequences^25–27^. Thirty mouse VH promoters (250 bp) were next chosen on the basis of their expression as measured by GRO-seq in resting and activated mouse B cells. VHHs were codon-optimized and complemented with mouse leading exons, introns and recombination signal sequences. The 30 units were pieced together by Gibson assembly (NEB) into the pBeloBAC11 vector.

### Embryonic stem cell targeting

E14 cells were cultured in Glasgow’s MEM (Thermo Fisher Scientific, 11710035) supplemented with 10% FBS (ATCC, SCRR-03-2020), Glutamax, sodium pyruvate, non-essential amino acids (NEAA), penicillin (50 units per ml)–streptomycin (50 μg ml^−1^) and β-mercaptoethanol (Thermo Fisher Scientific, 35050061, 11360070, 11140050, 15140122, 21985023, respectively) at 37 °C and 5% CO_2_. mLIF (GeminiBio, 400-495, 10,000×), MEKi (Stemgent, 0400602, 10,000×) and GSKi (Stemgent, 0400402, 3,333×) were added to the medium before use. Dishes and plates were coated with 0.1% glycine for 15 min at room temperature before use. To delete the CH1 exon of Cμ, sgRNAs targeting the flanking introns were cloned into the CRISPR–Cas9 plasmid pX458 (Addgene, 48138). A 100-nt-long single-stranded oligonucleotide (ODN) donor (100 μM, 3 μl) was co-transfected with the two Cas9 sgRNA plasmids (2 μg each) into embryonic stem (ES) cells (2 million cells) with the Amaxa nucleofection kit (Lonza, VPH-1001, programme A030). After 24 h of culture, GFP^high^ ES cells were FACS-sorted and cultured in 10-cm dishes at a concentration of 2,000 cells per dish. Seven days later, colonies were transferred into 96-well plates and cultured for an additional 3 days. Genomic DNA was then extracted and genotyped for Cμ exon deletion. Clones with homozygous deletions were selected to next delete the Cγ1 exon with the same strategy. To delete the entire VH locus, sgRNAs targeting sequences upstream of *Ighv1-86* (first Ighv) and downstream of *Ighv5-1* (last Ighv), respectively, were cloned into pX458. Selected ES cells (2 million cells) were co-transfected with the two Cas9 sgRNA plasmids (1.5 μg each) and the pLH28-exchange-cassette plasmid (1.5 μg) and then cultured in 10-cm dishes. Twenty-four h later, cells were selected with puromycin (0.8 μg ml^−1^) for 10 days and individual colonies were picked for expansion and genotyping by long-range PCR. Positive clones (2 million cells) were co-transfected with VHH minigene vector (3 μg) and a Cre-expressing plasmid (1 μg) and cultured in 10-cm dish for 3 days. Cells were then selected with ganciclovir (2 μg ml^−1^) for 7 days before individual colonies were picked for expansion and genotyping. sgRNAs and ODN primers are listed in Supplementary Table 4.

### Generation of nanomice

Two modified ES cell clones with normal karyotype were injected into C57BL/6 blastocysts, which were then transferred to the uteri of pseudopregnant C56BL/6 recipients. High-percentage chimeras were mated to C57BL/6 mice and offspring were genotyped for VHH minigene knock-in and Cμ and Cγ1 exon deletion. One out of three chimeras produced F_1_ offspring that showed germline transmission. Three F_1_ male mice were backcrossed with C57BL/6 mice. F_2_ heterozygous mice were inbred to produce mice homozygous for all three modifications. Two F_1_ offspring from the same chimera were null for Ch1 of *Ighm* but wild type for VH and CH1 exon of Cγ1. These mice were used as controls for Extended Data Fig. 3b.

### FACS analysis

B cells were activated by culturing them in RPMI 1640 supplemented with 10% FBS, HEPES, sodium pyruvate, NEAA, penicillin–streptomycin and β-mercaptoethanol at 37 °C and 5% CO_2_ in the presence of lipopolysaccharide (LPS, Sigma, L2630, 50 μg ml^−1^), interleukin-4 (IL-4, Sigma, I1020, 2.5 ng ml^−1^) and anti-CD180 (1:2,000, BD Pharmingen, 552128) antibody for 72 h. For proliferation assays, cells were stained with CellTracer Violet (Thermo Fisher Scientific, C34557) at room temperature for 20 min before culturing for 96 h. For all FACS staining, cells were incubated in FACS buffer (phosphate-buffered saline (PBS), 2% FBS) at 4 °C for 20 min. Antibodies used for staining were: anti-B220-PerCP–Cy5.5 (1:500, eBioscience, 45-045-82), anti-B220–APC (1:500, Invitrogen, 17-0452-83), anti-IgM–APC (1:500, eBioscience, 17-5790-82), anti-Igκ–PE (1:500, BD Pharmingen, 559940), anti-Igκ–FITC (1:500, BD Pharmingen, 550003), anti-Igλ–FITC (1:200, BD Pharmingen, 553434), anti-IgG1–PE (1:200, BD Pharmingen, 550083), anti-IgG1–APC (1:200, BD Pharmingen, 550874), anti-IgD–FITC (1:200, BD Pharmingen, 553439), anti-CD95–PE (1:200, BD Pharmingen, 554258), anti-CD43–PE (1:200, BD Pharmingen, 553271), anti-CD23–PE (1:200, BD Pharmingen, 553139), anti–CD21-FITC (1:200, Biolegend, 123408) and Viability Dye eFluor506 (1:1000, Invitrogen, 1923275). Data were acquired using BD FACSCanto and FACSDiva software and analysed with FlowJo software. Gating strategy is shown in Extended Data Fig. 10d.

### Analysis of VHH(D)J recombination

Genomic DNA from bone marrow or splenic samples was extracted with the DNeasy Blood & Tissue kit (Qiagen, 69506). VHH(D)J joints were PCR-amplified from 100 ng of DNA with a framework primer unique for each of the 30 VHHs, and a common downstream JH4 primer. PCR products were loaded onto 1% agarose gel to resolved them by size. Primers are listed in Supplementary Table 4.

### VHH(D)J recombinants phagemid library construction

VHH(D)J phagemid libraries from unimmunized mice were constructed by first extracting RNA from nanomouse splenic samples with Trizol (Thermo Fisher Scientific, 15596026) and reverse-transcribed to cDNA with SuperScript III (Thermo Fisher Scientific, 18080400) according to the manufacturer’s instructions with some modifications. Ten μg of total RNA was denatured and annealed with gene-specific primers corresponding to CH2 of *Ighm*. After elongation at 50 °C for 50 min, template-switching oligonucleotide (TSO) (3′-propyl modified) linker was added to the reaction and the first strand cDNA was elongated for another 90 min at 42 °C. The reaction was inactivated at 85 °C for 5 min and 2 μl of cDNA was used as template for VHH(D)J amplification by two-step PCR with HiFi PCR Premix (Takara, 639298). For the first-step PCR, unmodified TSO and *Ighm*-CH2-specific oligonucleotides were used. Thirty ng of the first-step PCR product was then amplified with a primer mix of 30 forward primers corresponding to framework (FR1) of 30 VHH genes and 4 reverse primers corresponding to JH1~JH4. pMES4 phagemid (Addgene, 98223) was amplified with primers to introduce SfiI sites on both ends. VHH(D)J and pMES4 fragments were then digested with SfiI (NEB, R0123L) and ligated (100 and 200 ng, respectively) with T4 ligase (NEB, M0202L) at 16 °C overnight. Ligation product was purified with DNA Clean & Concentrator (Zymo Research, D4014) and eluted into 12 μl of water. Three μl of DNA was electroporated into 60 μl of TG1 cells (Lucigen, 60502-2) in 1.0-mm cuvette (HARVARD Apparatus, 450134) with BTX electroporation system ECM 630 at the setting of 25 μF, 200 ohms, 1,600 volts. After 1 h recovery in 37 °C in shaker incubator, TG1 cells were plated on 5 of 10-cm LB agar plates supplemented with 100 μg ml^−1^ carbenicillin (KD Medical, BPL-2400). Plates were placed in 37-°C bacteria incubator overnight, bacteria were scraped off plates and phagemid library was DNA-extracted with Zymo Plasmid Miniprep kit (Zymo Research, D4054). Primers are listed in Supplementary Table 4.

### Sanger sequencing for somatic hypermutation analysis

VHH(D)J recombinants from splenic cells of two nanomice were PCR-amplified as described in ‘VHH(D)J recombinants phagemid library construction’, and then cloned directly into pCR-Blunt II-TOPO vector (Thermo Fisher Scientific, 450245) and transformed into Stabl3 competent *Escherichia coli* (Thermo Fisher Scientific, C737303). Ninety-six colonies were randomly picked for Sanger sequencing. TG1 cells from BG505 DS-SOSIP immunized nanomouse phagemid library were plated onto carbenicillin-containing plates and 50 colonies picked for Sanger sequencing. Sequence alignment was performed using Snapgene software.

### Immunizations

All animal-related procedures were performed by following our NIAMS ACUC protocol. To monitor the germinal centre reaction, three nanomice and two C57BL/6 mice were immunized intraperitoneally with 50 μg of keyhole limpet haemocyanin (KLH) in the presence of complete Freund’s adjuvant (CFA). A boost injection was performed in the footpads with 25 μg of KLH in the presence of incomplete Freund’s adjuvant (IFA) on day 6. Spleen samples were collected on day 12 for analysis.

To isolate nanobodies recognizing HIV-1 envelope trimer, one nanomouse was immunized intraperitoneally with 50 μg of BG505 DS-SOSIP in the presence of CFA on day 0, and boost immunized with 25 μg of BG505 DS-SOSIP in the presence of IFA or PBS on day 22 and 44, respectively. Bone marrow, spleen and blood were collected on day 48.

To isolate neutralizing nanobodies against SARS-CoV-2, a llama (Capralogics) was immunized subcutaneously with 1 mg of recombinant RBD protein in the presence of CFA at day 0, and boost immunized with 0.5 mg of RBD protein in the presence of IFA on days 14, 28 and 42. Two more boost immunizations with 0.5 mg of recombinant spike protein in the presence of IFA were performed on day 56 and 70. On day 80, 500 ml of whole blood were collected for library preparation.

To isolate SARS-CoV-2 neutralizing nanobodies from nanomice, two groups of mice (five for group 1 and six for group 2) were immunized with RBD and/or spike protein and bleeds were collected after a 62-day immunization protocol. Mice were immunized intraperitoneally with 50 μg of RBD protein (group 1) or spike protein (group 2) in the presence of CFA on day 0, and boost immunized intraperitoneally with 25 μg of RBD protein (group 1) or spike protein (group 2) in the presence of IFA on days 14, 28 and 42. Mice were further immunized with 25 μg of spike protein in PBS on day 56 and 59, intraperitoneally and intravenously, respectively. Bone marrow, spleen and blood samples were collected on day 62. The best responders—nanomouse 1 (group 1) and nanomice 2 and 3 (group 2)—were selected for phage library construction.

### Llama and nanomouse phage library construction

The llama phage library was constructed as previously described^28^ with some modifications. In brief, 300 ml of whole blood was collected from llama and peripheral blood mononuclear cells (PBMCs) were enriched using Ficoll-Paque plus (GE Healthcare, 17-1440-03). Fifty μg of extracted RNA was reverse-transcribed to cDNA with random hexamers and 2.5 μl of cDNA was used for first round RT–PCR with gene-specific primers CALL001 and CALL002. The PCR reaction was repeated in 12 individual tubes with cDNA added into reactions separately. PCR fragments of about 700-bp long were gel-purified and used as template (30 ng for each reaction, repeated in 12 individual tubes) for second round PCR with nested primers VHH-Back and VHH-For. PCR product from individual reactions were pooled and gel-purified. Nanobody fragments and pMES4 phagemid were digested with PstI-HF and BstEII-HF restriction enzymes (NEB: R3140L, R3162L) and ligated (1 μg and 2 μg respectively) with T4 ligase at 16 °C overnight. Ligation product was column-purified (into 12 μl of H_2_O) and electroporated into 360 μl of TG1 cells. After 1 h of recovery at 37 °C in a shaker incubator, cells were plated on 6 of 245 × 245-mm dish (Thermo Fisher Scientific, 431301) containing 2-YT agar supplemented with 100 μg ml^−1^ carbenicillin and 2% (w/v) glucose. Plates were placed in a 37-°C bacteria incubator overnight and then bacteria were scraped off of plates and archived as glycerol stocks. Cells were infected with VCSM13 helper phage (Agilent Technologies, 200251) followed by precipitation of culture supernatant with 20% polyethylene glycol 8000 (Sigma, 89510) in 2.5 M sodium chloride on ice to purify the nanobody phage particles. Phage particles were resuspended in 1 ml PBS, 300 μl were used for screening immediately and the remaining phages were stored at −80 °C in the presence of 10% glycerol.

Nanomouse nanobody phage libraries were constructed the same way as nanomouse VHH(D)J region phagemid library construction with some modifications. In brief, total RNA was extracted from splenic cells, bone marrow cells and PBMCs of immunized mice and processed separately until the TG1 cell electroporation step. RNA from splenic cells, bone marrow and PBMCs (50 μg, 50 μg and all, respectively) were reversed-transcribed to cDNA with *Ighg1*-CH2-specific primer in separate tubes. Two μl of cDNA was used as template for PCR variable domain amplification (12 reactions each), using unmodified TSO and *Ighg1*-CH2-specific oligonucleotide as primers. Second PCR was repeated in 12 reactions using 30 ng of the first-step PCR product as template and 30 FR1 and 4 JH oligonucleotide mix as primers. PCR products were gel-purified, digested with SfiI and ligated with pMES4 (200 ng and 400 ng, respectively). Ligation products from splenic cells, bone marrow and PBMC samples were pooled and column-purified (into 12 μl of water) and electroporated into 360 μl of TG1 cells and phage libraries prepared as described in ‘VHH(D)J recombinants phagemid library construction’. Primers are listed in Supplementary Table 4.

### Library construction for Illumina MiSeq deep sequencing

Phagemid DNA extracted from TG1 cell libraries was used as starting material for constructing MiSeq libraries to measure VHH use and nanobody diversity. In brief, 1.2 μg of phagemid DNA was used as template and VHH(D)J inserts were amplified with primers recognizing the pMES4 backbone using CloneAmp HiFi PCR Premix (Takara, 639298) in a 50-μl reaction (9 cycles). To avoid MiSeq failure owing to low complexity at initial cycles and to enable multiplex sequencing, 1–9-nt-long staggers were introduced into forward primers. Without purification, 5 μl of the first PCR product were used as template for a second PCR (9 cycles) to add Illumina P5 and P7 primers on both ends. PCR product was then loaded onto a 2% agarose gel and the approximately 580-bp size band was purified with Zymoclean Gel DNA Recovery Kit (Zymo Research, D4002). DNA concentration was determined by Qubit 4 Fluorometer (Thermo Fisher Scientific, Q33238) and average DNA size was determined by TapeStation 4150 (Agilent). DNA was then adjusted to 2 nM in elution buffer containing 0.1% Tween-20. For unimmunized nanomice VHH(D)J library, DNA (2 nM) from 3 mice was mixed at a 1:1:1 ratio and loaded for MiSeq run. For immunized llama and nanomice nanobody diversity analysis, DNA (2 nM) of pre-selection and post-selection libraries were mixed at 10:1 ratio first and then samples from individual animals were pooled at 1:1 ratio before loading for MiSeq sequencing. Primers are listed in Supplementary Table 4.

### Deep-sequencing analysis

For unimmunized nanomouse VHH use analysis, pooled library from 3 mice was sequenced by MiSeq (pair end, 270 cycles × 2). Pair-end reads were merged with NGmerge^29^ with default settings. Nucleotides corresponding to pMES4 were trimmed using pTrimmer program^30^, leaving clean VHH(D)J sequences in the merged reads. Reads with undetermined N nucleotides, low quality sequence or less than 300 nt in length were removed with the fastp program^31^. Fastq format sequences were converted to .fasta format for further analysis. To calculate VHH use, a BLAST database was built from a .fasta format file (vhh.exon.fa) containing the exon sequence of all 30 VHH genes, using BLAST+. VHH(D)Js were then aligned to VHH genes using igblast program^32^. The alignment output file was simplified to retain only sequence identifier and VHH(D)J recombination information.

For immunized llama and nanomouse nanobody diversity analysis, in total 8 libraries (pre- and post-selection) were sequenced by MiSeq (pair end, 300 cycles × 2). The 3′-end low-quality sequences were trimmed using the Sickle program (v1.33, available at https://github.com/najoshi/sickle). For different libraries, the minimum length of trimmed sequence was adjusted on the basis of the length of staggers in the primers used for library construction. Paired sequences were merged by flash program (v.1.2.11)^33^ and translated. To extract nanobody sequences and to locate CDR3 regions, we used ANARCI program^34^ to annotate VHH genes with IMGT numbering. Protein sequences with greater than or equal to 100 amino acids in total and greater than or equal to 1 amino acid in the CDR3 region were extracted for further analysis. Enrichment of individual sequences were calculated by comparing their frequencies in pre- and post-selection libraries. Sequences that were enriched more than 10 times and had greater than or equal to 5 × 10^−5^ frequency were selected for CDR3 clustering using cd-hit program (v.4.6.8)^35^.

### Expression and purification of BG505 DS-SOSIP and SARS-CoV-2 proteins

BG505 DS-SOSIP protein was expressed and purified as previously described^36^. The spike protein of SARS-CoV-2 and its RBD were expressed and purified as previously described^37,38^ with some modifications. In brief, 1 mg of pCAGGS-Spike or pCAGGS-RBD plasmid was transfected into 1 l of Expi293 cells (Thermo Fisher Scientific, A14528) with Turbo293 transfection reagent (Speed Biosystem, PXX1002). Supernatants from transfected cells were collected on day 4 after transfection by centrifugation of the culture at 12,000*g* for 15 min. Supernatant was then filtered through 0.2 m aPES filter (Thermo Fisher Scientific, 5670020) and incubated with 10 ml of cOmplete His-tag purification resin (Roche, 05893801001) for 1 h at room temperature. Next, His-tag resin was collected through gravity flow columns (BioRad, 9704652), washed with 100 ml of washing buffer (15 mM imidazole, 50 mM TrisHCl, 300 mM NaCl) and eluted with 25 ml of elution buffer (300 mM imidazole, 50 mM TrisHCl, 300 mM NaCl). Eluate was concentrated in 10-kDa Amicon Centrifugal Units (EMD Millipore, UFC901024) and then dialysed in PBS using Slide-A-Lyzer dialysis cassette (Thermo Fisher Scientific, 66381). Proteins were analysed by NuPAGE gel (Thermo Fisher Scientific, NP0336BOX) and visualized by InstantBlue staining (Abcam, ab119211). Soluble spike trimers or monomeric RBD proteins were aliquoted, snap-frozen by liquid nitrogen and stored at −80 °C before being used for immunization. RBD and spike (HexaPro) proteins used for phage screening, BLI, negative-stain electron microscopy and cryo-electron microscopy (cryo-EM) were done as previously described^10,39^.

### Phage screening for BG505 DS-SOSIP, RBD and spike binding nanobodies

RBD, spike and BG505 DS-SOSIP were coated by different methods onto MaxiSorp 96-well plate (Thermo Fisher Scientific, 439454) for phage screening. For RBD screening, two wells were coated with 50 μl of RBD protein (100 μg ml^−1^ in PBS) at 4 °C overnight. Another well with 50 μl of PBS was used as an non-coated control. Wells were washed with PBS with 0.1% Tween-20 three times and blocked with 5% non-fat milk in PBS at room temperature for 1 h. For spike or BG505 DS-SOSIP screening, three wells were coated with 50 μl of lectin (EMD Millipore, L8275, 100 μg ml^−1^ in PBS) at 4 °C overnight. Wells were washed and blocked with 10% non-fat milk in room temperature for 1 h. After three washes, 50 μl of 100 μg ml^−1^ of BG505 DS-SOSIP or spike were added to 2 wells, incubated at room temperature for 2 h and washed. A third well contained PBS and served as a non-coated control. Three hundred μl of phage particles was mixed with 300 μl of 10% non-fat milk and rotated gently at room temperature for 1 h. One hundred and fifty μl of blocked phage particles was then added into each well and incubated in room temperature for 2 h with gentle shaking. After 15 washes, phages were eluted with TrypLE Express Enzyme (Thermo Fisher Scientific, 12605010) by shaking plates at 700 rpm at room temperature for 30 min and used immediately for selection efficiency estimation (10 μl of phage eluate) and recovery infection (the remaining eluate) as previously described^28^. Anti-RBD libraries were selected with RBD protein once, and libraries constructed from BG505 DS-SOSIP or spike immunized animals were selected with BG505 DS-SOSIP or spike (HexaPro) proteins twice.

### Enzyme-linked immunosorbent assay selection of anti-BG505 DS-SOSIP and anti-RBD nanobodies

After one or two rounds of selection, recovered TG-1 cells were plated and colonies were picked to prepare periplasmic extracts containing crude nanobodies for enzyme-linked immunosorbent assay (ELISA). In brief, individual colonies were picked and grown in 96 deep-well plates (Thermo Fisher Scientific, 278743) in 2YT medium supplemented with 100 μg ml^−1^ of carbenicillin and 0.1% glucose. IPTG (final 1 mM) was added when optical density at 600 nm (OD_600_) reached 1 and protein expression was induced in 30 °C for 16 h. Periplasmic extracts were prepared by resuspending bacteria pellet in 200 μl of PBS and rapidly frozen in liquid nitrogen. Frozen cells were thawed slowly at room temperature and centrifuged at 4,100*g* for 15 min. Maxisorp plates were coated with lectin (2 μg ml^−1^) followed by BG505 DS-SOSIP (2 μg ml^−1^) or with RBD (2 μg ml^−1^). After blocking, 100 μl of nanobody-containing supernatant were transferred to the plates and incubated for 2 h at room temperature. Plates were washed and then incubated with horse radish peroxidase (HRP) conjugated goat anti-alpaca VHH domain specific antibody (Jackson ImmunoResearch, 128-035-232) for 1 h at room temperature. Plates were washed and then developed by addition of 50 μl of tetramethylbenzidine (TMB) (Thermo Fisher Scientific, 34028) for 10 min, then the reaction was stopped by adding 50 μl of 1 M H_2_SO_4_. Absorbance at 450 nm was measured with Synergy microplate reader (BioTek Gen5).

### Expression and purification of nanobodies

Phagemids from lead candidates identified by ELISA were extracted from TG-1 cells and transformed into WK6 cells (ATCC, 47078). Cultures were grown in 30 ml of 2YT medium (100 μg ml^−1^ of carbenicillin and 0.1% glucose) at 37 °C and 220 rpm until OD_600_ reached 1. Protein expression was induced by 1 mM IPTG at 30 °C for 16 h and then pelleted at 4,100*g* for 15 min. The resulting pellets were resuspended in 1 ml of PBS plus 30 μl of 0.5 MU ml^−1^ polymyxin B (Sigma, P1004) and incubated at 37 °C with shaking for 1 h. Cell debris were pelleted at 12,000*g* for 5 min and nanobodies in the supernatant were purified using Capturem His-tagged purification kit (Takara, 635710). For larger-scale nanobody production (0.2 to 1 l of culture), nanobodies in the supernatant were purified by cOmplete His-tag purification resin and dialysed in PBS as described in ‘Expression and purification of BG505 DS-SOSIP and SARS-CoV-2 proteins’. Proteins were filtered sterile by 0.22-μm PVDF membrane (EMD Millipore, UFC30GVNB) before being used for downstream assays.

### Expression and purification of Fc conjugated nanobodies and RBD in Expi293 cells

Monomeric or trimeric nanobody sequences were fused to the Fc region of human IgG1 with 6×His tag at the C-terminal end and cloned into the pVRC8400 vector. In trimeric form, nanobody units were connected through (GGGGS)×3 flexible linkers. In some cases, llama IgG2a hinge region was used in lieu of human IgG1 hinge. The Fc fusion constructs were expressed in Expi293 cells as described in ‘Expression and purification of BG505 DS-SOSIP and SARS-CoV-2 proteins’ at 33 °C from day 2 to day 4. Antibodies in the supernatant were purified using either cOmplete His-tag purification resin or protein A (Thermo Fisher Scientific, A26457). When protein A resin was used, antibodies were eluted by IgG elution buffer (Thermo Fisher Scientific, 21009) and brought to neutral pH by adding 1/10 volume of Tris-HCl (1M, pH 8). Antibodies were concentrated, dialysed and filtered. Nanobody–Fc yields were up to 100 mg l^−1^. RBD region of SARS-CoV, SARS-CoV-2 and bat coronavirus WIV16 spike protein were fused to the Fc region of human IgG1 and cloned into pVRC8400 vector. RBD–Fc proteins were expressed in Expi293 cells and purified with protein A.

### SARS-CoV-2 surrogate virus neutralization test

RBD–ACE2 interaction blocking potential of nanobodies was tested using the SARS-CoV-2 surrogate virus neutralization test (sVNT) kit (Genscript, L00847) according to the manufacturer’s instructions. In brief, HRP–RBD was diluted and incubated with specified concentrations of nanobodies for 30 min at 37 °C. Samples were then transferred onto ACE2-coated plates and incubated for 15 min at 37 °C. Plates were washed, and the assay was developed using TMB reagent and quenched with stop solution. Absorbance at 450 nm was measured with a Synergy microplate reader (BioTek Gen5). Inhibition rate was calculated and plotted using Microsoft Excel according to manufacturer’s instruction of the sVNT kit.

### Pseudotyped virus neutralization assay

A panel of plasmids expressing RBD-mutant SARS-CoV-2 spike proteins in the context of pSARS-CoV-2-SD19 have previously been described^1,40^. The mutants E484K and KEN (K417N, E484K and N501Y) were constructed in the context of a pSARS-CoV-2-S_Δ19_ variant with a substitution in the furin cleavage site (R683G). The IC_50_ of these pseudotypes were compared to a wild-type SARS-CoV-2 spike sequence carrying R683G in the subsequent analyses, as appropriate. Generation of SARS-CoV-2 pseudotyped HIV-1 particles and pseudovirus neutralization assay was performed as previously described^11^. In brief, 293T cells were transfected with pNL4-3DEnv-nanoluc and pSARS-CoV-2-SD19 and pseudotyped virus stocks were collected 48 h after transfection, filtered and stored at −80 °C. Serially diluted nanobodies were incubated with the pseudotyped virus for 1 h at 37 °C. The mixture was added to 293T_ACE2_^11^ (for analysis of wild-type neutralization activity) (Fig. 2) or HT1080Ace2 cl.14^17^ cells (for analysis of spike mutant panel) (Fig. 3), and after 48 h cells were washed with PBS and lysed with Luciferase Cell Culture Lysis 5x reagent (Promega). Nanoluc luciferase activity in lysates was measured using the Nano-Glo Luciferase Assay System (Promega) with Modulus II Microplate Reader User interface (TURNER BioSystems). The relative luminescence units were normalized to those derived from cells infected with SARS-CoV-2 pseudotyped virus in the absence of antibodies. Neutralization of HIV-1-based SARS-CoV-1 and bat coronavirus WIV16 pseudotypes were performed in HT1080/ACE2cl.14 cells as previously described^41^. The IC_50_ for nanobodies was determined using four-parameter nonlinear regression (GraphPad Prism).

Recombinant Indiana vesicular stomatitis virus (rVSV) expressing different coronavirus spikes (SARS-CoV-2, RaTG13, GDPangolin, GXPangolin, SARS-CoV, WIV1, SHC014, LYRa11, Rs7327, Rs4084 and Rs4231) were generated as previously described^2,42,43^. In brief, HEK293T cells were grown to 80% confluency before transfection with the spike gene using Lipofectamine 3000 (Invitrogen). Cells were cultured overnight at 37 °C with 5% CO_2_, and VSV-G pseudo-typed ΔG-luciferase (G*ΔG-luciferase, Kerafast) was used to infect the cells in DMEM at a multiplicity of infection (MOI) of 3 for 2 h before washing the cells with 1× DPBS three times. The next day, the transfection supernatant was collected and clarified by centrifugation at 300*g* for 10 min. Each viral stock was then incubated with 20% I1 hybridoma (anti-VSV-G, ATCC: CRL-2700) supernatant for 1 h at 37 °C to neutralize contaminating VSV-G pseudo-typed ΔG-luciferase virus before measuring titres and making aliquots to be stored at −80 °C. Neutralization assays were performed by incubating pseudoviruses with serial dilutions of antibodies and scored by the reduction in luciferase gene expression as previously described^2,42,43^. In brief, 293T_ACE2_ cells were seeded in 96-well plates (2 × 10^4^ cells per well). Pseudoviruses were incubated with serial dilutions of the antibodies in triplicate for 30 min at 37 °C. The mixture was added to cultured cells and incubated for an additional 16 h. Luminescence was measured using Luciferase Assay System (Promega), and IC_50_ was defined as the dilution at which the relative light units were reduced by 50% compared with the virus control wells (virus + cells) after subtraction of the background in the control groups with cells only. The IC_50_ values were calculated using a five-parameter dose–response curve in GraphPad Prism.

### Authentic SARS-CoV-2 microplate neutralization

The SARS-CoV-2 viruses USA-WA1/2020 (WA1), USA/CA_CDC_5574/2020 (B.1.1.7), hCoV-19/South Africa/KRISP-EC-K005321/2020 (B.1.351) and hCoV-19/Japan/TY7-503/2021 (P.1) were obtained from BEI Resources (NIAID, NIH) and propagated for one passage using Vero E6 cells. Virus infectious titre was determined by an end-point dilution and cytopathic effect (CPE) assay on Vero E6 cells as previously described^42^. An end-point dilution microplate neutralization assay was performed to measure the neutralization activity of nanobodies. In brief, nanobodies were subjected to successive fivefold dilutions starting from 10 μg ml^−1^. Triplicates of each dilution were incubated with SARS-CoV-2 at an MOI of 0.1 in EMEM with 7.5% inactivated fetal calf serum for 1 h at 37 °C. After incubation, the virus–nanobody mixture was transferred onto a monolayer of Vero E6 cells grown overnight. The cells were incubated with the mixture for about 70 h. CPE of viral infection was visually scored for each well in a blinded fashion by two independent observers. The results were then reported as percentage of neutralization at a given nanobody dilution. The IC_50_ for nanobodies was determined using nonlinear regression (normalized response, variable slope) in GraphPad Prism.

### Nanobody stability studies

Nanobody was nebulized with a portable mesh nebulizer (Philips, InnoSpire Go) producing 2-5 μm particles at a final concentration of 0.4 mg ml^−1^. The resulting aerosol was collected by condensation into a 50-ml tube cooled on ice. Pre- and post-nebulization samples were analysed by NuPAGE gel and visualized by InstantBlue staining. SARS-CoV-2 surrogate virus neutralization test was also performed to compare the neutralization potency of pre- and post-nebulization samples. For thermostability tests, nanobodies supplemented with loading buffer (Thermo Fisher Scientifc, NP0007) and β-mercaptoethanol were heated at 98 °C for 10 min and then analysed on a NuPAGE gel and visualized by InstantBlue staining.

### BLI assay to measure nanobody affinity

The BLI assay was performed using a fortéBio Octet Red384 instrument to determine the affinity of nanobodies to RBD. In brief, biotinylated-RBD was immobilized onto streptavidin-coated biosensors and then dipped into a solution containing the nanobody for 30 s followed by dissociation for 2–3 min. To assay the binding of nanobodies to RBD–Fc (SARS-CoV, SARS-CoV-2 and WIV16), 6×His-tagged nanobody was immobilized onto Ni-NTA coated biosensors and then dip into RBD–Fc solution for association for 1 min followed by dissociation for 1 min. Sensorgrams of the concentration series were corrected with corresponding blank curves and fitted globally with Octet evaluation software using a 1:1 Langmuir model of binding.

### Nanobody–RBD binding competition assay

Nanobody–RBD binding competition assay was performed using a fortéBio Octet Red384 instrument. Biotinylated-RBD was first immobilized onto streptavidin coated biosensors and allowed to associate with one of the six nanobodies, then dipped into a solution contained a second nanobody.

### Negative-staining electron microscopy analysis for the structure of nanobody–spike complex

Nanobody–spike complexes were prepared by mixing the two proteins at a 1:1 weight ratio, then diluted with a buffer containing 10 mM HEPES, pH 7.4, 150 mM NaCl, adsorbed to a freshly glow-discharged carbon-coated copper grid, washed with the above buffer, and stained with 0.75% uranyl formate. Images were collected at a magnification of 57,000 using EPU on a Thermo Fisher Talos F200C microscope equipped with a 4k × 4k CETA 16 M camera and operated at 200 kV. The pixel size was 2.5 Å for the CETA camera. Particle picking, reference-free 2D classification, 3D reconstruction and refinement were performed using cryoSPARC.

### Cryo-EM data collection and processing

Nanobody–spike complexes (Nb12–S6P and Nb30–S6P) were prepared by manual mixture of the two proteins in a 1:1 weight ratio, then diluted to a final concentration of 0.5 mg ml^−1^. Samples (2.7 μl) were applied to a glow-discharged Quantifoil R 2/2 gold grids and vitrified using a Vitrobot Mark IV with a blot time of 3 s before the grid was plunged into liquid ethane. Data were acquired using the Leginon system installed on Titan Krios electron microscopes operating at 300 kV and equipped with a K3-BioQuantum direct detection device. The dose was fractionated over 40 raw frames and collected over a 2-s exposure time. Motion correction, CTF estimation, particle picking, 2D classifications, ab initio model generation, heterogeneous refinements, 3D variability analysis and homogeneous 3D refinements were carried out with cryoSPARC. Local refinement was performed to resolve the RBD–nanobody interface by using a mask encompassing one copy of the RBD–nanobody complex for refinement, after removing the rest of the density by particle subtraction.

### Cryo-EM model fitting

For initial fits to the cryo-EM reconstructed maps, we used the coordinates of the SARS-CoV-2 spike from Protein Data Bank (PDB) code 7JZL, and a nanobody model predicted by the ABodyBuilder server^44^. These initial models were docked into the cryo-EM maps using Chimera. The coordinates were then fit to the electron density more precisely through an iterative process of manual fitting using Coot and real space refinement within Phenix, Molprobity and EMRinger were used to check geometry and evaluate structures at each iteration step. Figures were generated in UCSF ChimeraX and PyMOL (https://pymol.org). Map-fitting cross correlations were calculated using Fit-in-Map feature in UCSF Chimera. Overall and local resolution of cryo-EM maps was determined using cryoSPARC.

### Informatics analysis

Sequence entropy are based on nine strains with the following UniProt identifiers: SARS-CoV-2: P0DTD1, B.1.1.7 and B.1.351; SARS-CoV: A7J8L4, Q202E5 and P59594; and bat SARS-like coronavirus: MG772933, WIV16 (A0A0U2IWM2) and RsSHC014 (U5WLK5). The entropy was calculated for each residue based on aligned sequences with the formula:

$${\rm{Entropy}}\,=-\mathop{\sum }\limits_{i=1}^{21}p(xi)\log (p(xi))$$

In which *xi* are standard amino acids, plus gap.

The buried surface area on the RBD were calculated for 51 human antibody–SARS-CoV-2 RBD complexes using the Naccess program.

### Data presentation

Figures arranged in Adobe Illustrator 2020.

### Reporting summary

Further information on research design is available in the Nature Research Reporting Summary linked to this paper.

## Online content

Any methods, additional references, Nature Research reporting summaries, source data, extended data, supplementary information, acknowledgements, peer review information; details of author contributions and competing interests; and statements of data and code availability are available at 10.1038/s41586-021-03676-z.

## Supplementary information

Supplementary Figure 1 Original, uncropped images of the gels used in Extended Data Fig. 2b and Extended Data Fig. 7.

Reporting Summary

Supplementary Table 1 Raw data used to calculate SHM pre- and post-immunization (shown in Fig. 1d) and numbers of VHHs obtained by deep-sequencing used to create the bar graph shown in Extended Data Fig. 2c.

Supplementary Table 2 Data collection and refinement statistics for cryoEM experiments.

Supplementary Table 3 Surface area of RBD residues buried by 51 human antibodies.

Supplementary Table 4 A list of all oligos used in the current study.

Supplementary Table 5 The raw data used to calculate SHM pre- and post-immunization (shown in Fig. 1d) and numbers of VHHs obtained by deep-sequencing used to create the bar graph shown in Extended Data Fig. 2c.

## Data Availability

Raw data and original images are provided in Supplementary Table 5 and Supplementary Fig. 1. The accession numbers for the deep-sequencing data reported in this Article can be found at GSE167310. Coordinates and maps for reported cryo-EM structures have been deposited in the Electron Microscopy Data Bank and PDB at EMD-24078 and EMD-24077, and 7MY3 and 7MY2, respectively. Any other relevant data are available from the corresponding authors upon reasonable request.
