# Supplementary material for: Nanobodies from camelid mice and llamas neutralize SARS-CoV-2 variants
Source: Nature. 2021 Jun 7;595(7866):278–82. doi: 10.1038/s41586-021-03676-z (PMC8260353; doi:10.1038/s41586-021-03676-z)
Supplement: Supplementary file 1 — Original, uncropped images of the gels used in Extended Data Fig. 2b and Extended Data Fig. 7. [file 41586_2021_3676_MOESM1_ESM.pdf]

nature portfolio

https://doi.org/10.1038/s41586-021-03676-z

Supplementary information

Nanobodies from camelid mice and llamas neutralize SARS-CoV-2 variants

In the format provided by the authors and unedited

Original source image of Extended Data Figure 2b

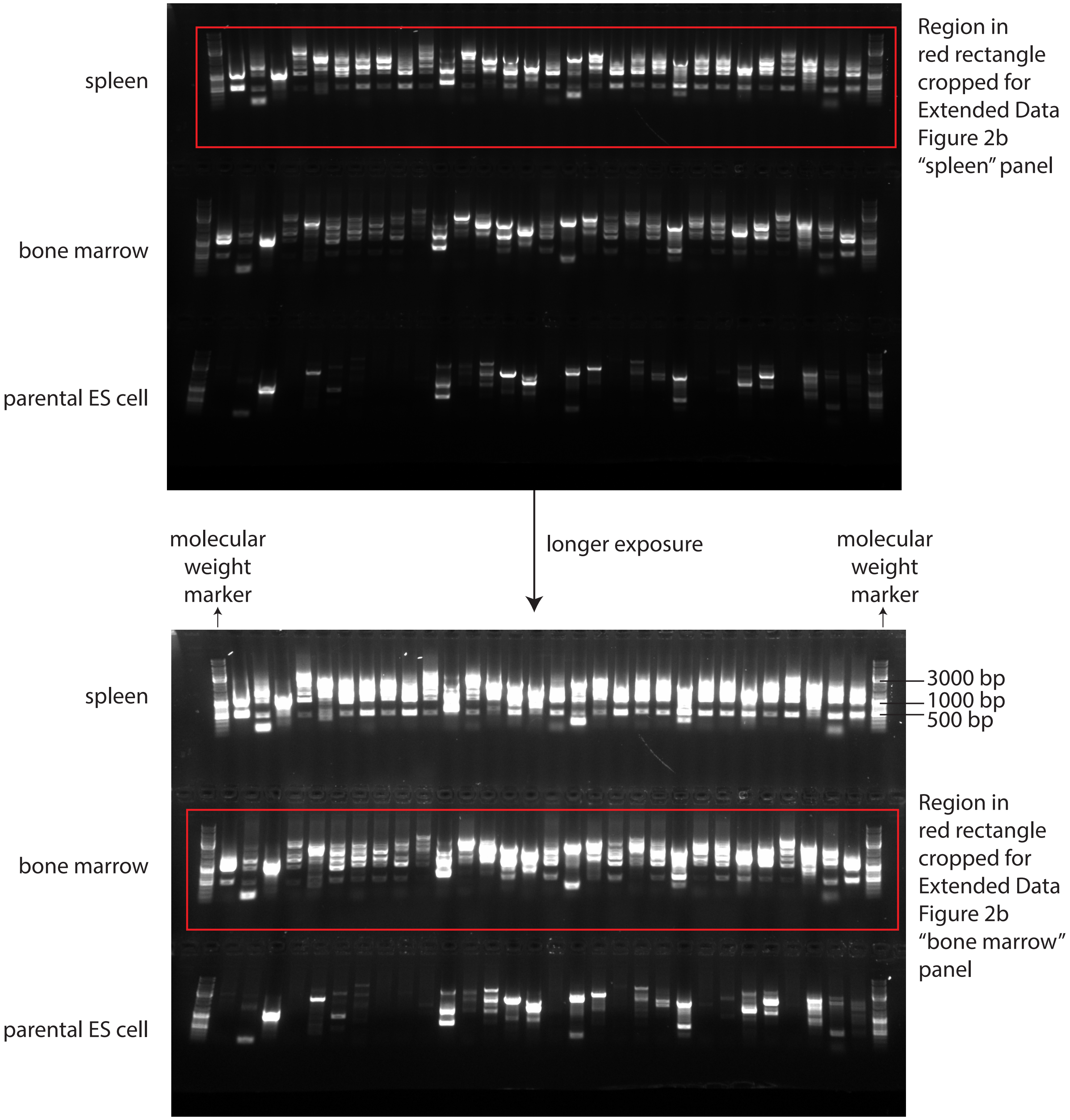

Original source image of Extended Data Figure 7

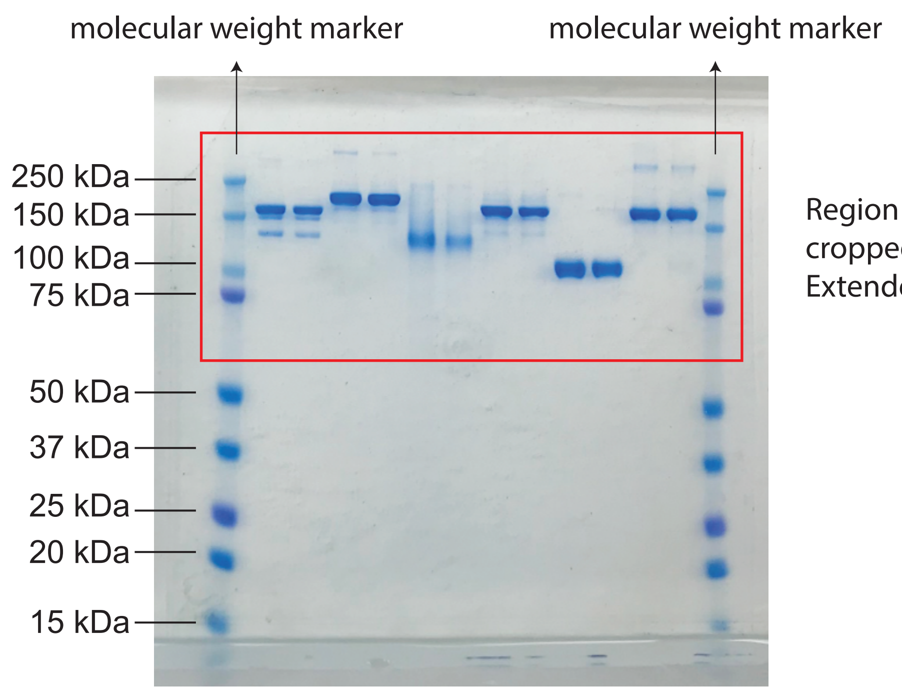

Region in red rectangle cropped for Extended Data Figure 7b

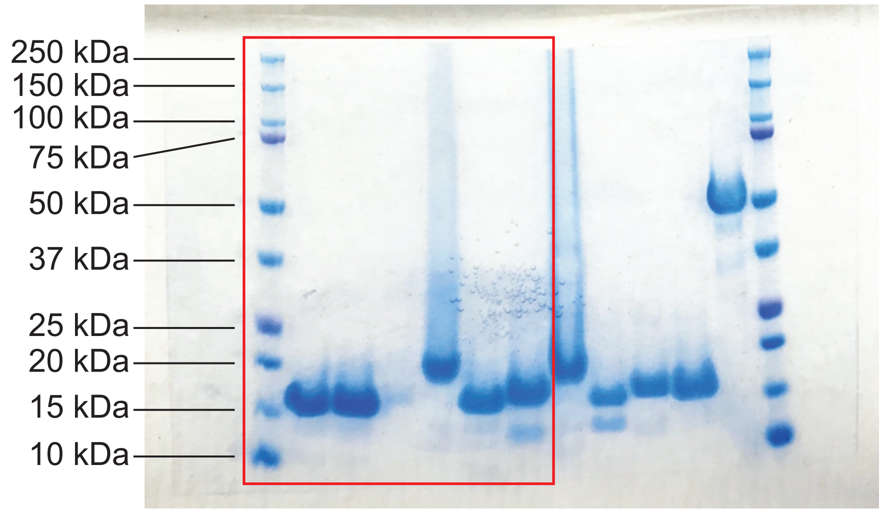

Region in red rectangle cropped for Extended Data Figure 7d left panel

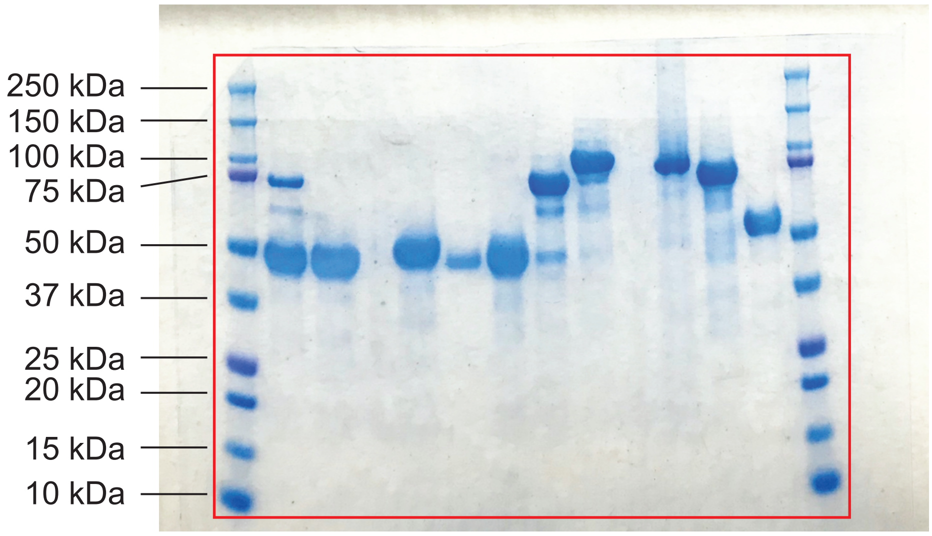

Region in red rectangle cropped for Extended Data Figure 7d right panel
