# Supplementary material for: Nanobodies from camelid mice and llamas neutralize SARS-CoV-2 variants
Source: Nature. 2021 Jun 7;595(7866):278–82. doi: 10.1038/s41586-021-03676-z (PMC8260353; doi:10.1038/s41586-021-03676-z)
Supplement: Supplementary file 2 — Reporting Summary [file 41586_2021_3676_MOESM2_ESM.pdf]

nature research

Corresponding author(s): Jianliang Xu, Michel Nussenzweig, Peter D Kwong, Rafael Casellas

Last updated by author(s): Apr 29, 2021

Reporting Summary

Nature Research wishes to improve the reproducibility of the work that we publish. This form provides structure for consistency and transparency in reporting. For further information on Nature Research policies, see our Editorial Policies and the Editorial Policy Checklist.

Statistics

For all statistical analyses, confirm that the following items are present in the figure legend, table legend, main text, or Methods section.

n/a	Confirmed	
☐	☒	The exact sample size (*n*) for each experimental group/condition, given as a discrete number and unit of measurement
☐	☒	A statement on whether measurements were taken from distinct samples or whether the same sample was measured repeatedly
☒	☐	The statistical test(s) used AND whether they are one- or two-sided<br>*Only common tests should be described solely by name; describe more complex techniques in the Methods section.*
☒	☐	A description of all covariates tested
☒	☐	A description of any assumptions or corrections, such as tests of normality and adjustment for multiple comparisons
☐	☒	A full description of the statistical parameters including central tendency (e.g. means) or other basic estimates (e.g. regression coefficient) AND variation (e.g. standard deviation) or associated estimates of uncertainty (e.g. confidence intervals)
☒	☐	For null hypothesis testing, the test statistic (e.g. *F*, *t*, *r*) with confidence intervals, effect sizes, degrees of freedom and *P* value noted<br>*Give P values as exact values whenever suitable.*
☒	☐	For Bayesian analysis, information on the choice of priors and Markov chain Monte Carlo settings
☒	☐	For hierarchical and complex designs, identification of the appropriate level for tests and full reporting of outcomes
☒	☐	Estimates of effect sizes (e.g. Cohen's *d*, Pearson's *r*), indicating how they were calculated

Our web collection on statistics for biologists contains articles on many of the points above.

Software and code

Policy information about availability of computer code

Data collection	BD FACSDiva Software version 9.0 for FACS; Modulus II Microplate Reader User interface version 2.1.0 by TURNER BioSystems; BioTek Gen 5 software for ELISA; Leginon system installed on Titan Krios electron microscopes for CryoEM; MiSeq software v3.1 for Miseq.
Data analysis	FlowJo 10.7.1 for FACS analysis; GraphPad Prism 8.4.2; Microsoft Excel 16.36; Snapgene 4.2.11 for sequence analysis; Fortebio Octet Data Analysis Software 8.0; cryoSPARC 2.15 for EM analysis; NGmerge version 0.2, pTrimmer version 1.3.3, fastp version 2020, BLAST+ version 2.11.0, IgBLAST version 1.17.0, Sickle v1.33, FLASH v1.2.11, ANARCI version 2019 and CD-HIT v4.6.8 for deep sequencing analysis.

For manuscripts utilizing custom algorithms or software that are central to the research but not yet described in published literature, software must be made available to editors and reviewers. We strongly encourage code deposition in a community repository (e.g. GitHub). See the Nature Research guidelines for submitting code & software for further information.

Data

Policy information about availability of data

All manuscripts must include a data availability statement. This statement should provide the following information, where applicable:

- Accession codes, unique identifiers, or web links for publicly available datasets
- A list of figures that have associated raw data
- A description of any restrictions on data availability

All sequencing data are deposited at GEO (GSE167310). All structural data are deposited at EMDB and PDB (EMD-24078, EMD-24077, PDB-7MY3 and PDB-7MY2).

Field-specific reporting

Please select the one below that is the best fit for your research. If you are not sure, read the appropriate sections before making your selection.

☒ Life sciences ☐ Behavioural & social sciences ☐ Ecological, evolutionary & environmental sciences

For a reference copy of the document with all sections, see nature.com/documents/nr-reporting-summary-flat.pdf

Life sciences study design

All studies must disclose on these points even when the disclosure is negative.

Sample size	To ensure good immune response be obtained, five and six nanomice were immunized with SARS-CoV-2 RBD and Spike recombinant protein, respectively. Best responders were used for nanobody library construction. Ten llamas were screened for serum antibody titer against rabies and clostridium vaccine, and then one llama with highest titer was immunized with RBD and Spike recombinant protein.
Data exclusions	Best responder mice, one from RBD immunized group, two from Spike immunized group, were picked for nanobody isolation. The rest of mice were not used for further analysis. Exclusion criteria were not pre-established.
Replication	All experiments successfully repeated at least twice.
Randomization	This is not relevant as this is an observational study and there is no selection or accidental bias introduced in the study.
Blinding	Real SARS-CoV-2 virus neutralization assay on WA1, B.1.1.7, B.1.351 and P.1 was performed double blinded. For all other experiments, investigators were not blinded during data collection and analysis, as blinding was not relevant in those observational studies where no bias was introduced.

Reporting for specific materials, systems and methods

We require information from authors about some types of materials, experimental systems and methods used in many studies. Here, indicate whether each material, system or method listed is relevant to your study. If you are not sure if a list item applies to your research, read the appropriate section before selecting a response.

Materials & experimental systems

n/a	Involved in the study	
☐	☒	Antibodies
☐	☒	Eukaryotic cell lines
☒	☐	Palaeontology and archaeology
☐	☒	Animals and other organisms
☒	☐	Human research participants
☒	☐	Clinical data
☒	☐	Dual use research of concern

Methods

n/a	Involved in the study	
☒	☐	ChIP-seq
☐	☒	Flow cytometry
☒	☐	MRI-based neuroimaging

Antibodies

Antibodies used	anti-B220-PerCP-Cy5.5 (eBioscience, 45-045-82), anti-B220-APC (Invitrogen, 17-0452-83), anti-IgM-APC (eBioscience, 17-5790-82), anti-Igk-PE (BD Pharmingen, 559940), anti-Igk-FITC(BD Pharmingen, 550003), anti-Igl-FITC (BD Pharmingen, 553434), anti-IgG1-PE (BD Pharmingen, 550083), anti-IgG1-APC (BD Pharmingen, 550874), anti-IgD-FITC (BD Pharmingen, 553439), anti-CD95-PE (BD Pharmingen, 554258), anti-CD43-PE (BD Pharmingen, 553271), anti-CD23-PE (BD Pharmingen, 553139), anti-CD21-FITC (Biolegend, 123408), Viability Dye eFluor506 (Invitrogen, 1923275), anti-VHH (Jackson ImmunoResearch, 128-035-232), anti-CD180 (BD Pharmingen, 552128)
Validation	All antibodies are commercially available with at least one reference citation.

Eukaryotic cell lines

Policy information about cell lines

Cell line source(s)	E14 ES cells (ATCC CRL-1821)<br>293T (ATCC CRL-11268)<br>293TAce2 (derived from 293T); was generated in the Laboratory of Retrovirology, Rockefeller University (Dr. Paul D. Bieniasz)<br>VeroE6 (ATCC CRL-1586)<br>Expi293 (Thermo Fisher Scientific, A14528)<br>WK6 cells (ATCC, 47078)

Authentication	Not authenticated after purchase.
Mycoplasma contamination	All cell lines tested negative for mycoplasma contamination.
Commonly misidentified lines (See ICLAC register)	No commonly misidentified cell lines were used.

Animals and other organisms

Policy information about studies involving animals; ARRIVE guidelines recommended for reporting animal research

Laboratory animals	Five (2 male and 3 female, 10 weeks old) and six (4 male and 2 female, 8 weeks old) nanomice were immunized with SARS-CoV-2 RBD and Spike recombinant protein, respectively. One llama (male, 2 years old) was immunized with RBD and Spike recombinant protein.
Wild animals	The study did not involve wild animals.
Field-collected samples	The study did not involve samples collected from the field.
Ethics oversight	Animal study was approved by NIAMS ACUC at ht the NIH.

Note that full information on the approval of the study protocol must also be provided in the manuscript.

Flow Cytometry

Plots

Confirm that:

☒ The axis labels state the marker and fluorochrome used (e.g. CD4-FITC).

☒ The axis scales are clearly visible. Include numbers along axes only for bottom left plot of group (a 'group' is an analysis of identical markers).

☒ All plots are contour plots with outliers or pseudocolor plots.

☒ A numerical value for number of cells or percentage (with statistics) is provided.

Methodology

Sample preparation	Cells isolated from bone marrow, spleen or peritoneal cavity of nanomice were washed with PBS before staining. Cells stimulated in culture medium for 72-96 hours were also washed with PBS before staining.
Instrument	FACSCanto (Becton Dickinson)
Software	BD FACSDiva Software and FlowJo
Cell population abundance	Cells were stained and analyzed on FACSCanto directly, no sorting applied.
Gating strategy	Cells were first gated for lymphocytes in FSC-A (x-axis) versus SSC-A (y-axis). We identify single cells in FSC-W versus FSC-H, and then SSC-W versus SSC-H. We then select B220+ Live B Cells (Viability Dye eFluor506 negative, Invitrogen) for further analysis.

☒ Tick this box to confirm that a figure exemplifying the gating strategy is provided in the Supplementary Information.
